# Mechanistic Insights into Electrocatalytic Hydrogen Evolution by an Exceptionally Stable Cobalt Complex

**DOI:** 10.1021/acs.inorgchem.4c01043

**Published:** 2024-04-19

**Authors:** Maria B. Brands, Joost N. H. Reek

**Affiliations:** Homogeneous, Supramolecular and Bio-inspired Catalysis, Van ‘t Hoff Institute for Molecular Sciences, University of Amsterdam, Science Park 904, 1098 XH Amsterdam, The Netherlands

## Abstract

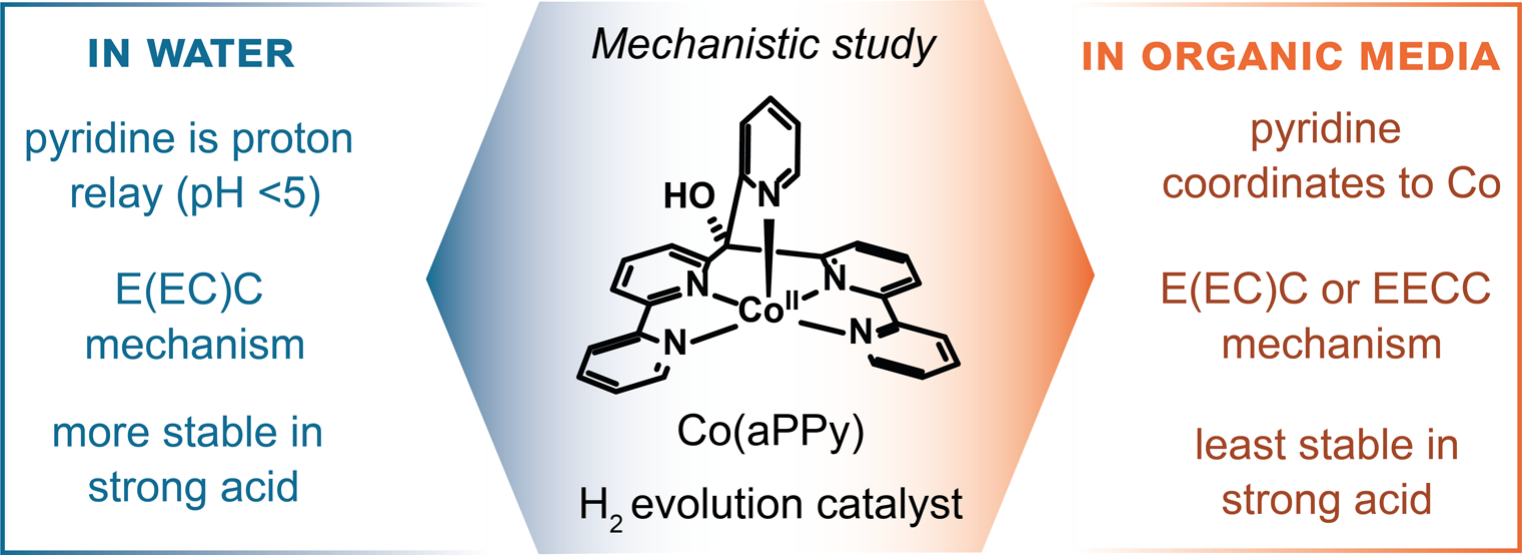

Co(aPPy) is one of the most stable and active molecular first-row transition-metal catalysts for proton reduction reported to date. Understanding the origin of its high performance via mechanistic studies could aid in developing even better catalysts. In this work, the catalytic mechanism of Co(aPPy) was electrochemically probed, in both organic solvents and water. We found that different mechanisms can occur depending on the solvent and the acidity of the medium. In organic solvent with a strong acid as the proton source, catalysis initiates directly after a single-electron reduction of Co^II^ to Co^I^, whereas in the presence of a weaker acid, the cobalt center needs to be reduced twice before catalysis occurs. In the aqueous phase, we found drastically different electrochemical behavior, where the Co(aPPy) complex was found to be a precatalyst to a different electrocatalytic species. We propose that in this active catalyst, the pyridine ring has dissociated and acts as a proton relay at pH ≤ 5, which opens up a fast protonation pathway of the Co^I^ intermediate and results in a high catalytic activity. Furthermore, we determined with constant potential bulk electrolysis that the catalyst is most stable at pH 3. The catalyst thus functions optimally at low pH in an aqueous environment, where the pyridine acts as a proton shuttle and where the high acidity also prevents catalyst deactivation.

## Abstract 2

The development of stable and active hydrogen evolution catalysts based on abundant metals is challenging. Via electrochemical studies, we provide insights into the conflicting earlier reported mechanisms of the HER by the exceptionally stable and active Co(aPPy) complex, which may lead to knowledge-guided design of new catalysts. We propose that Co(aPPy) only in water has a dissociated pyridine ligand that functions as a proton relay and observe higher catalyst stability at low pH.

## Introduction

Hydrogen formation via water splitting is a promising endeavor to produce a clean, carbon-free energy carrier,^1−4^ provided that the required energy for this reaction is obtained from a sustainable source such as wind or sunlight. The best hydrogen evolution catalysts (HECs) are currently based on platinum;^5^ however, considering the price and abundance, the development of catalysts based on earth-abundant metals is key. The progress of the past decade in this field has resulted in interesting candidates, such as nanostructured metal phosphides like CoP,^6^ and alloyed first-row transition metals, including NiMO_4_/MoO_2_@Ni.^7^ Although these materials show great promise, their performance can still be improved in terms of the activity and stability. The design of improved catalysts has proven to be challenging as the predictability of the reactivity of these materials is currently limited. Besides catalytic materials, studies have also focused on molecular HECs, as the performance of these catalysts is relatively easy to design and tune via the ligand environment. HECs based on Fe, Co, and Ni show promising results^8−12^ and owe part of their success to their ligand environment, either by optimizing the electronic properties of the metal center or by providing beneficial functionalities in the second coordination sphere, such as proton relays. Although water should ideally be used as solvent and proton source, these molecular catalysts are commonly employed in organic solvents due to their limited solubility or poor stability in water. Since stable, water-tolerating HECs are an absolute necessity for large-scale hydrogen production, it is of great importance to improve the stability of molecular catalysts in the presence of water.^3^

**Figure 1:**
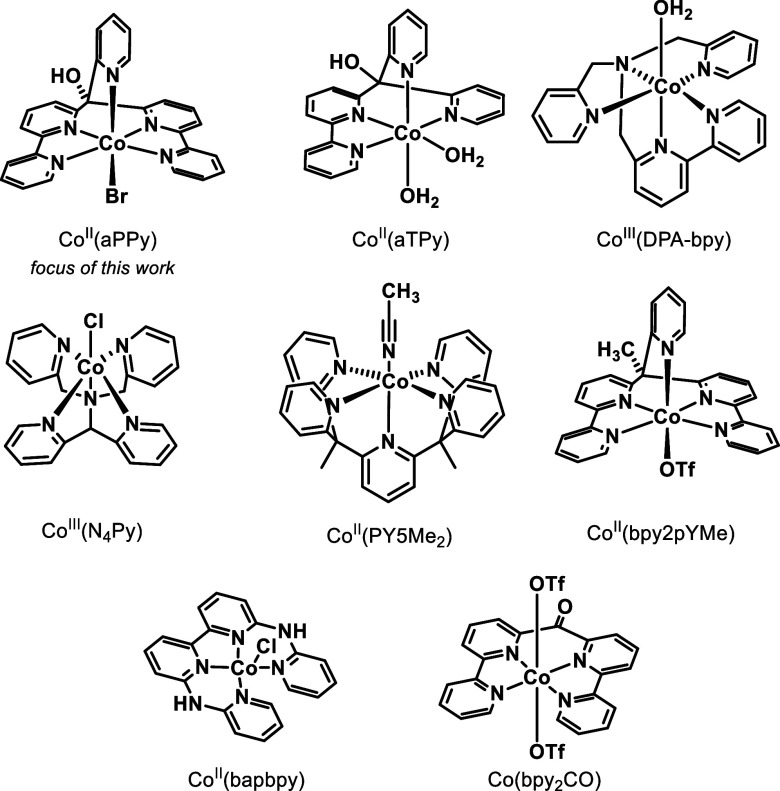
Various reported Co^II^-polypyridyl catalysts for proton reduction.

Cobalt polypyridyl complexes show relatively high stabilities in aqueous environments (Figure 1).^13−22^ Within this class, Co(aPPy) is one of the most stable catalysts and consists of a Co^II^ metal center surrounded by the *aPPy* ligand (short for “alternative penta-pyridine”). The ligand consists of two bipyridine rings, bridged in the 2′-position via a carbon atom, which contains a hydroxy group and another pyridine ring connected at the 2′-position. The five pyridines then coordinate to the cobalt ion, resulting in a geometrically distorted complex. This distortion is proposed to be one of the reasons for the high activity of Co(aPPy).^23^

The multidentate ligand provides a stable binding pocket for the Co metal center, and earlier photocatalytic studies have found that the Co(aPPy) can reach record turnover numbers (TONs) up to 33,300 (in 11 h) and is most stable between pH 4.5 and 5.5.^21,23,25^ Similar multidentate Co^II^ complexes are still outperformed by Co(aPPy) in terms of stability and activity under the same conditions (Table SI5).^19,21^ Therefore, we are interested in investigating whether there are any additional properties of Co(aPPy) that result in its exceptional performance.

**Figure 2:**
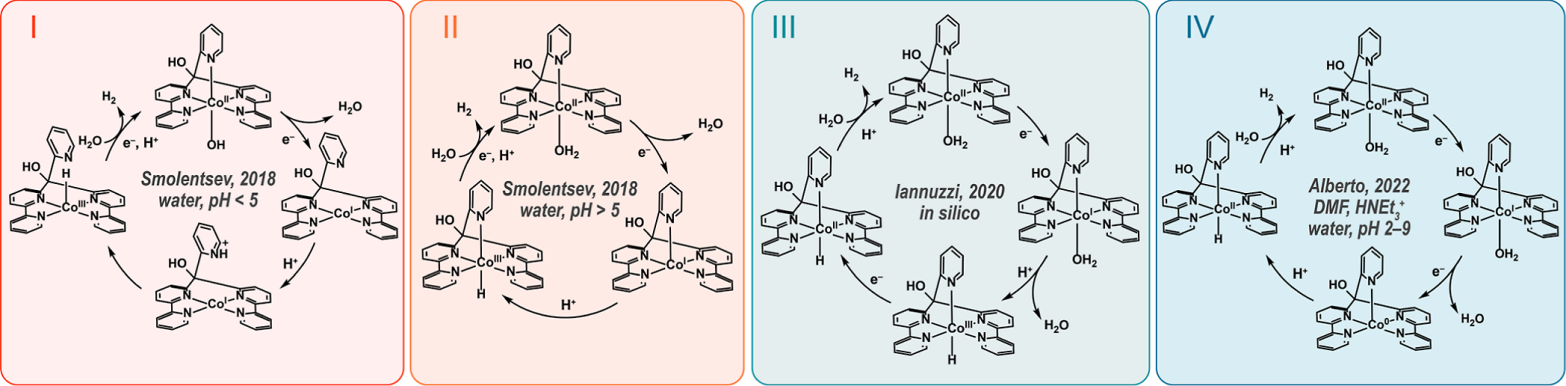
Various reported mechanisms for Co(aPPy) adapted from the literature—mechanistic details are discussed in the text. (I) Proposed mechanism by Smolentsev et al.^20^ in an aqueous environment below pH 5 and (II) above pH 5. (III) Calculated mechanism by Iannuzzi and Gurdal.^24^ (IV) Proposed mechanism by Alberto and co-workers, both in organic (HNEt_3_^+^ as an acid source) and aqueous media (pH 2–9).^19^

A better understanding of the relation between structure and performance can help to further improve the design of future first-row transition-metal HECs. Mechanistic studies have proven to be very insightful in this regard, leading to the identification of key steps and intermediates in the catalytic cycle. Currently, the mechanism of Co(aPPy) is still under debate, as the four previously proposed mechanisms show some disparities (Figure 2).^19,20,24^ First, Smolentsev et al.^20^ propose two possible pathways, depending on the pH of the solution. At low pH, the pyridine ligand potentially dissociates and acts as a proton relay, protonating the reduced Co^I^ center. On the other hand, at pH > 5, the pyridine would remain coordinated, and the Co^III^–H intermediate is obtained via direct protonation of Co^I^ (Figure 2II). In both mechanisms, after electron transfer and protonation of the Co^III^–H intermediate, hydrogen is formed and the initial complex is reobtained. The third option (Figure 2III), as calculated by Iannuzzi and Gurdal,^24^ follows a similar path as that of Smolentsev et al. at pH > 5, only herein, the Co^III^–H intermediate is reduced and protonated stepwise. Finally, as a fourth possibility, the group of Alberto recently proposed a mechanism where the initial Co^II^ is reduced twice, followed by two protonations, which generates H_2_ and yields the complex back in its original state.^19^

All four mechanisms are accompanied by experimental or theoretical support, but so far, explanations for their mutual differences and similarities have not been pinpointed yet. In this study, we report that some of these different mechanisms are in fact related, and we propose several additions to the possible catalytic pathways. By electrochemically probing the catalyst under several conditions,^16,19^ we hypothesize that different mechanisms can prevail, depending on the applied conditions. Furthermore, we report how these different mechanisms affect the catalyst performance during bulk electrolysis in aqueous media.

## Results and Discussion

The [Co(aPPy)Br]Br complex was synthesized according to an adjusted literature procedure (see Supporting Information), resulting in an increased yield and scale compared to previously reported work.^23^ We characterized the complex by UV–vis spectroscopy (Figure SI11), single-crystal X-ray diffraction (Figure SI12), high-resolution mass spectrometry (Figure SI10), nuclear magnetic resonance spectroscopy (Figure SI9), electron paramagnetic resonance spectroscopy (Figure SI13), and UV–vis spectroelectrochemistry (Figures SI14 and SI15). The results are in agreement with previously reported characterization data of Co(aPPy) and analogous complexes.^22,23,26^ After the successful synthesis, we studied the complex by electrochemical experiments.

### Electrochemistry of the Co(aPPy) Complex

**Figure 3:**
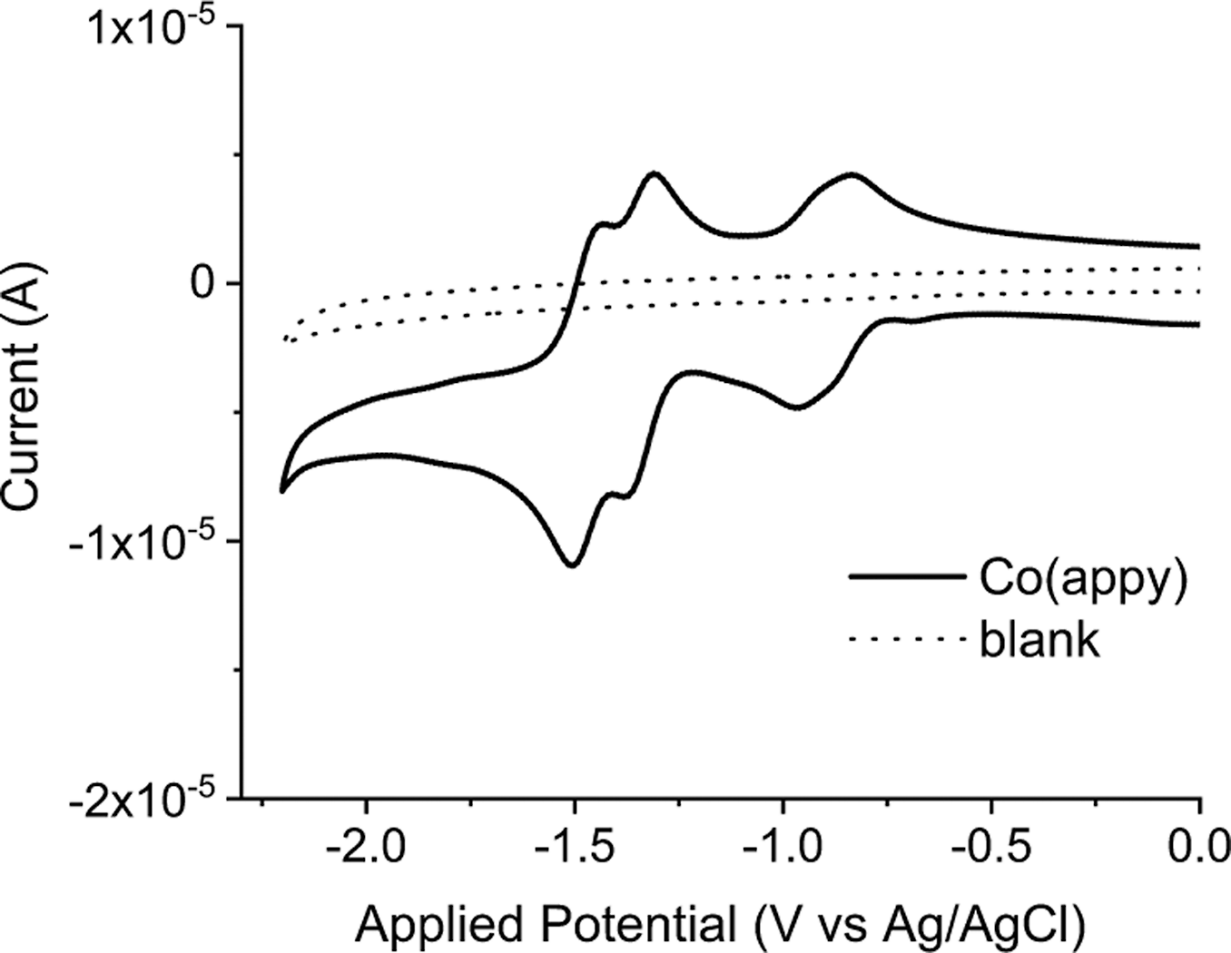
Cyclic voltammogram (IUPAC plotting convention) of [Co(aPPy)Br]Br (1 mM) in dry DMF (0.1 M TBABF_4_) purged with Ar, WE = glassy carbon, CE = Pt wire (*d* = 0.5 mm), and RE = Ag/AgCl (in 3 M KCl) in an undivided cell, without IR drop compensation. The second scans are shown, starting at 0.0 V, scanning toward cathodic potential with ν = 0.1 V·s^–1^.

Co(aPPy) was initially studied in dimethylformamide (DMF, Figure 3), via cyclic voltammetry (CV) in the absence of a substrate. The CV of the dissolved [Co(aPPy)Br]Br complex was similar to that of a Co(aPPy) analogue with noncoordinating perchlorate anions (Figure SI24), indicating that the halide ligand dissociated or exchanged for a solvent molecule.^17,27,28^ The complex itself shows multiple chemically reversible redox events: the redox couple at an *E*_1/2_ of −0.8 V (vs Ag/AgCl) was attributed to the Co^II/I^ couple, and the two events at −1.3 and −1.5 V were earlier reported as ligand-based reductions.^19,22^ These latter redox events were earlier proposed to occur on the bpy ligands, as similar electrochemical behavior was observed for the Zn(bpy)_2_pyMe analogue, which contains a redox-inactive metal center at these potentials.^22^ As noted by Long and co-workers, the influence of the bpy ligands on the redox properties of the complex is 2-fold: (i) charge is delocalized from the reduced Co^I^ center to the bpy ligands, likely through π-backbonding and (ii) at more negative potentials, the bpy ligands function as electron storage sites.^22^

### Proton Reduction by Co(aPPy) in Organic Media

Next, we studied the catalytic activity of Co(aPPy) for proton reduction in the presence of various acids. Since the acid strength can have a significant influence on the preferred catalytic pathway, the reduction of HBF_4_ (p*K*_a_ = 3.45^29^), HNEt_3_BF_4_ (p*K*_a_ = 9.25^30^), and acetic acid (p*K*_a_ = 13.5^31^) was investigated.^16^ Acetic acid was eventually not included in this study as the changes in all redox events suggest that acetate binds to the complex (see Figure SI19). Acetate binding has been observed before for similar Co^II^ complexes^14^ and alters the structural and electronic properties to such an extent that it becomes hard to compare the results to the catalytic reduction of other acids.

**Figure 4:**
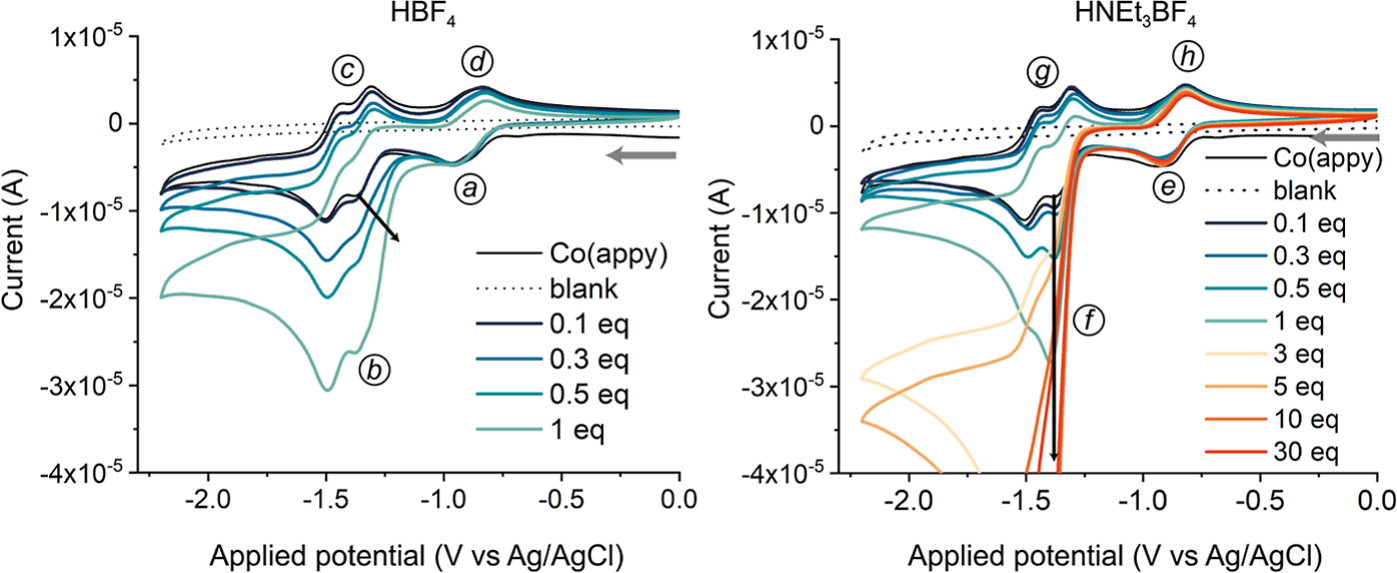
Cyclic voltammograms (IUPAC plotting convention) of [Co(aPPy)Br]Br (1 mM) in dry DMF (0.1 M TBABF_4_) purged with Ar, WE = glassy carbon, CE = Pt wire (*d* = 0.5 mm), and RE = Ag/AgCl (in 3 M KCl) in an undivided cell, without IR drop compensation. The second scans are shown, starting at 0.0 V, scanning toward cathodic potential with ν = 0.1 V·s^–1^ (indicated by the gray arrows). The equivalents of acid are given with respect to the catalyst. Left: increasing [HBF_4_] as a proton source. Right: increasing [HNEt_3_BF_4_] as a proton source.

The cyclic voltammograms in the presence of HBF_4_ as a proton source are shown in Figure 4 (left panel). Upon an increase in the concentration of HBF_4_, the reduction at −1.0 V (*a*) remained unchanged. The second reduction wave (*b*) at −1.3 V showed a current enhancement with an [acid]-dependent onset potential. The corresponding oxidation peak disappeared simultaneously (*c*), whereas the oxidation of Co^I^ to Co^II^ remained unaffected (*d*). The current increase at the second reduction wave, accompanied by a loss of reversibility of this redox couple, indicates that proton reduction catalysis occurs at these potentials. The associated onset potential shifts anodically with increasing [HBF_4_] (black arrow), which can be indicative of a protonation between the first and second reduction or of an energetic coupling between the reduction and the protonation.^16^ Finally, the presence of the Co^I^ oxidation peak in the back scan indicates that Co^I^ is an intermediate in the catalytic cycle. These combined results suggest that proton reduction catalysis occurs via an *E*(*EC*)*C* mechanism, where *E* stands for a one-electron reduction, (*EC*) for a reduction and protonation that are energetically coupled, and *C* is for a protonation.

In the presence of the weaker acid HNEt_3_BF_4_ as a proton source (Figure 4, right panel), the voltammograms again showed that the Co^II/I^ reduction remained unaltered (*e*). A strong current enhancement was observed after further reduction of the Co^I^ complex (*f*), with an onset potential of −1.20 V that was independent of the acid concentration. The catalytic current was accompanied by a loss of reversibility of the Co^I^ reduction (*g*), whereas Co^II/I^ oxidation remained unaffected (*h*). With the catalysis initiating after the second reduction, and with the presence of Co^I^ in the back scans, these results indicate that catalysis likely occurs via the stepwise *EECC* mechanism.^19^

Further increasing the acid concentration resulted in a loss of the redox features in CV. For HBF_4_, this occurred after adding more than 1 equivalent (see Figure SI20), and for HNEt_3_BF_4_, the peaks were lost after the addition of 30 equiv. The loss of the redox features is indicative of catalyst decomposition, of which the exact pathway is currently unknown. Possibly, decomposition proceeds via multiple ligand-based protonations, which can be detrimental for the stability of Co-polypyridyl complexes, as it leads to dissociation of the cobalt ion.^19,32,33^ This possible explanation is in line with Co(aPPy) withstanding relatively higher concentrations of the weaker acid before it decomposes.

### Proton Reduction in Water: A Different Active Species

**Figure 5:**
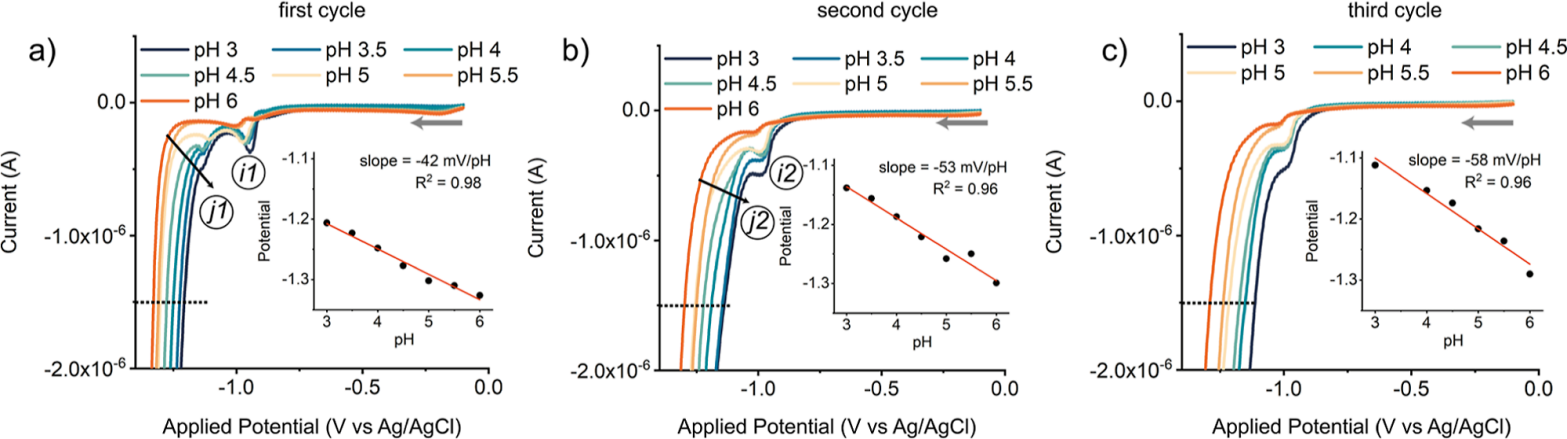
Forward scans of the cyclic voltammograms (IUPAC plotting convention) of the (a) first, (b) second, and (c) third scan of [Co(aPPy)Br]Br (1 mM), measured at varying pH in Milli-Q (0.2 M citric acid, 0.1 M NaBF_4_) purged with Ar. As electrodes, WE = HMDE, CE = carbon rod, and RE = Ag/AgCl (in 3 M KCl) were used in an undivided cell, without IR drop compensation. Starting at −0.1 V, the potential was swept toward cathodic potential with ν = 0.1 V·s^–1^. The insets show the pH dependency of the potentials measured at −1.5 × 10^–6^ A (indicated with the black dotted lines) for each respective scan.

**Figure 6:**
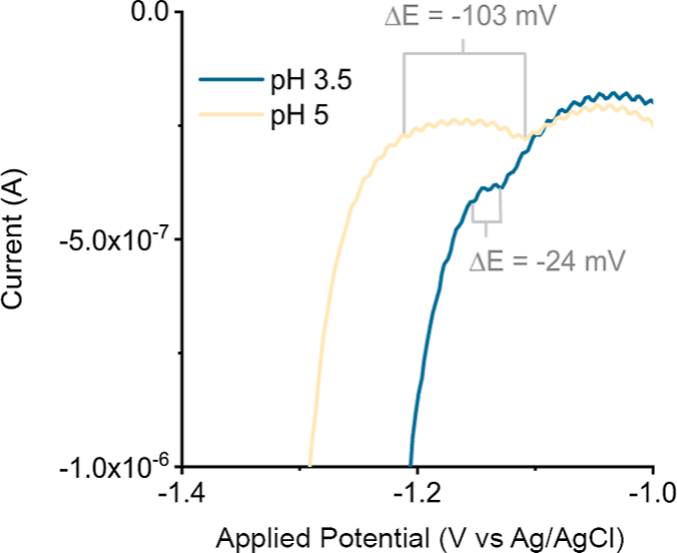
Zoom-in on the forward scans of the cyclic voltammograms (IUPAC plotting convention) of the first scan of [Co(aPPy)Br]Br (1 mM) at pH 3.5 and 5 in Milli-Q (0.2 M citric acid, 0.1 M NaBF_4_, purged with Ar). The difference between the potential of the prewave and the catalytic onset potential (Δ*E*) is indicated with the gray brackets. As electrodes, WE = HMDE, CE = carbon rod, and RE = Ag/AgCl (3 M KCl) were used in an undivided cell, without IR drop compensation. Starting at −0.1 V, the potential was swept toward cathodic potential with ν = 0.1 V·s^–1^.

Ideally, water is the proton source for hydrogen evolution; hence, the Co(aPPy) complex was further studied in aqueous media. We evaluated several buffer systems (including phosphate, Britton–Robinson, and ascorbate; see Supporting Information) and found that citric acid is the most suitable (pH 3–6.2).^21,25,34^ We thus measured the cyclic voltammograms of 1 mM Co(aPPy) in 0.1 M citric acid solution, with 0.1 M NaBF_4_ as the electrolyte in Milli-Q. In a typical experiment, three subsequent cycles were measured, between 0 and −1.7 V (vs Ag/AgCl). The pH was varied in the different experiments from pH 3 to 6, to probe the effect of the acidity on the mechanism. We used a hanging drop mercury electrode (HMDE) as the working electrode, due to its high overpotential for proton reduction in water, thereby reducing background activity. The resulting voltammograms are shown in Figures 5 and 6 (forward scans) and 7 (backward scans). Since we observed significant changes in the consecutive CV cycles, the first, second, and third cycle are plotted and discussed independently.

Scanning toward negative potential in the first cycle (Figure 5, left panel), the pH-independent reduction of Co^II^ to Co^I^ was observed (−0.96 V, step *i*1), followed by a prewave at −1.13 V (step *j*1), and a strong current enhancement at more cathodic potentials. Such a prewave can be indicative of the conversion of a precatalytic species into the active electrocatalyst and is thus extremely relevant to the catalytic mechanism.^35^ These results suggest the conversion of Co(aPPy) to a different catalyst because: (1) the prewave is electrochemically irreversible and absent in subsequent cycles, (2) the catalytic onset potential moves toward anodic potential after multiple scans (Figure 5, *j*2), and (3) line crossing occurs between the forward and backward scan in the first cycle (Figure SI25).

Therefore, we examined the nature of this precatalyst conversion further, via inspection of the potential difference (Δ*E*) between the prewave (*j*1) and the catalytic onset potential, as illustrated in Figure 6. At pH 3.5, catalysis initiated almost immediately after the prewave with a relatively small Δ*E* of −24 mV. Δ*E* increased at higher pH, as at pH 5, the difference had become −103 mV. An increasing Δ*E* is indicative of a slower conversion of the precatalyst into the active catalyst since more time is needed to complete the transformation, while the potential sweep continues. Since the Δ*E* increased with pH, precatalyst conversion is thus faster under acidic conditions, which suggests that a protonation is involved. The Co(aPPy) complex in water thus probably converts to the active catalyst via reduction and protonation.

Further clues on the identity of the electrocatalytic species could be obtained from the pH dependency of the potential at a fixed catalytic current (Figure 5, dashed line at −1.5 μA), which indicates coupling of the redox process to a protonation.^36^ In the first scan, this potential shows a linear pH dependency of −42 mV/pH (Figure 5, left panel, inset). This dependency became steeper in the subsequent cycles: in the second and third scan, the slope increased to −53 and −58 mV/pH, respectively (Figure 5, insets). The steepening slope thus implies that the influence of the protonation on the electron transfer changes over multiple scans.^27,36^ Overall, these results point toward an *E*(*EC*)*C* mechanism.

**Figure 7:**
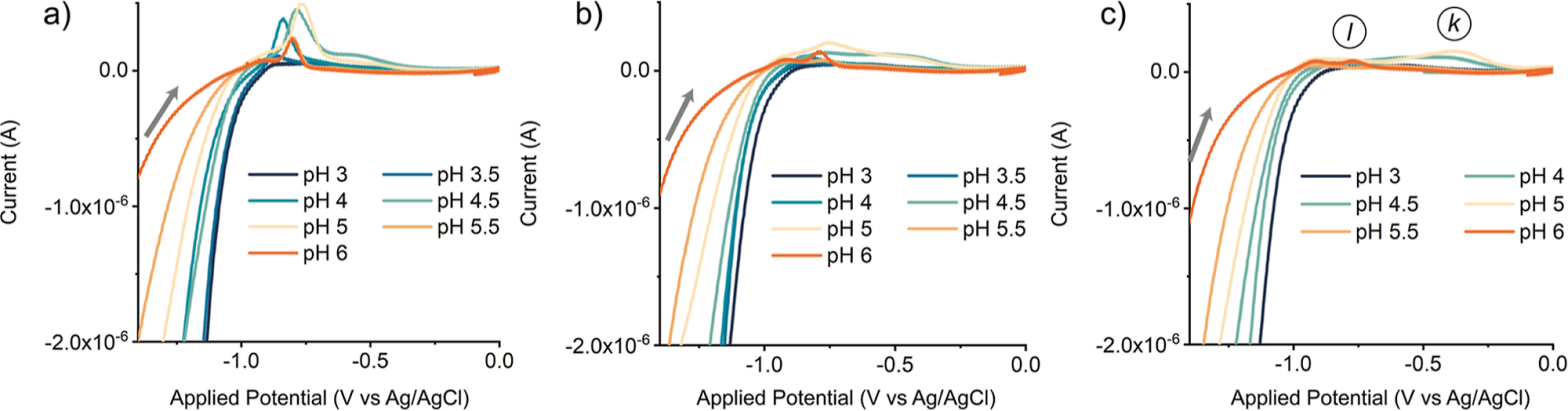
Backward scans of the cyclic voltammograms (IUPAC plotting convention) of the (a) first, (b) second, and (c) third scan of [Co(aPPy)Br]Br (1 mM), measured at varying pH in Milli-Q (0.2 M citric acid, 0.1 M NaBF_4_) purged with Ar. As electrodes, WE = HMDE, CE = carbon rod, and RE = Ag/AgCl (in 3 M KCl) were used in an undivided cell, without IR drop compensation. The backward scan started at −1.7 V, and the potential was swept toward anodic potential with ν = 0.1 V·s^–1^ (gray arrows).

Finally, we examined the oxidation peaks in the back scan, which can provide information about key catalytic intermediates. A complex mixture of oxidation peaks was observed in the first reverse scan (Figure 7a), but after three cycles, the oxidations converged to more defined peaks (Figure 7b,c). Their varying nature at different pH values can be divided into three categories. First, at pH < 4, no oxidation events were observed, indicating that the catalytic intermediates are transient and either (1) decompose, (2) can only be oxidized outside of this potential window, or (3) have quickly reacted away via protonation. At these highly acidic conditions, we view the latter scenario as most likely. Second, between pH 4.5 and 5, a broad feature was observed around −0.3 V (Figure 7c, *k*). Broad features like these have been reported before for Co^II^-catalyzed proton reduction as cobalt hydride species^37^ and have been identified as hydride intermediates for other HECs as well.^35^ These broad peaks thus likely indicate the presence of a relatively stable Co–H intermediate. Finally, at pH > 5, an oxidation event was found at −0.8 V (Figure 7c, *l*), which we attribute to the oxidation of a Co^I^ intermediate, based on the similar feature observed in organic media (Figure 4, points *d* and *h*). Protonation of the Co^I^ intermediate thus seems relatively slow at pH > 5, which allows for its detection in the back scan. At pH 4.5–5, the protonation of Co^I^ is too fast to observe this intermediate, but the lifetime of the hydride is long enough to observe it. This shift around pH 5 in which intermediates were detected hints toward the involvement of the pyridine ligand, as the change in detectable intermediates coincides with the p*K*_a_ of the ligand (5.25,^38^ see also section SI11).

Based on these combined results, we propose that the active catalytic species is generated from the Co(aPPy) complex via (1) dissociation of the axial pyridine ligand and (2) protonation of this group at pH < 5.5 and (3) it remains dissociated during catalysis. The pyridinium group could then act as a proton relay, facilitating fast protonation of the Co^I^, making this intermediate unobservable in the back scan at pH < 5.5. These claims are further supported by the mechanistic studies of Smolentsev et al.^20^ In addition, proton relays are known to have a potential beneficial effect on the catalytic performance of cobalt polypyridyl HECs,^15,39,40^ which would explain why the earlier reported record TONs of Co(aPPy) are highest at pH 5, close to the p*K*_a_ of the pyridine.^38^ Additional control experiments were performed to demonstrate that the active species is homogeneous in nature (Randles–Sevcik analysis, Figure SI26), that the catalyst is mononuclear, and that the reaction is first order in the catalyst (Figure SI27).

### Overview of the Proposed Mechanisms

**Scheme 1:**
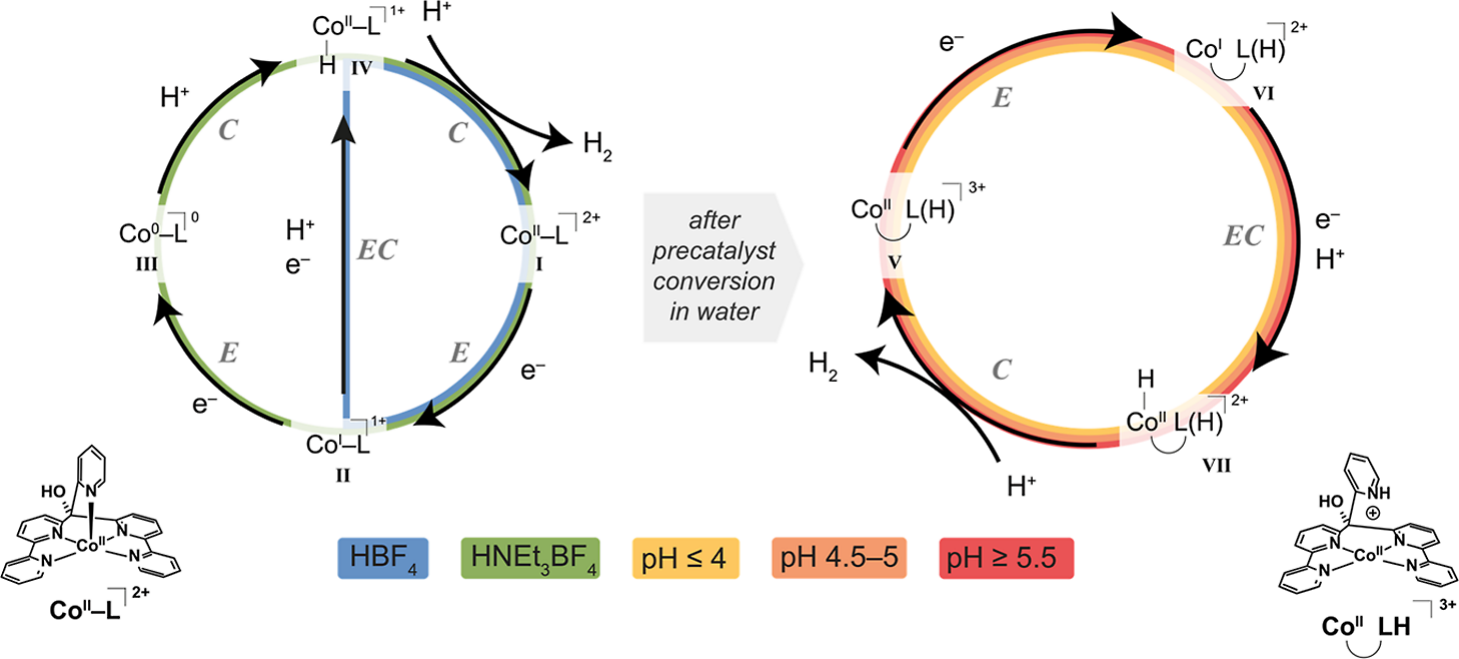
Proposed Mechanistic Pathways under Various Conditions Left: catalytic pathways in organic solvents. Right: catalytic pathway in an aqueous environment at pH 3–6, after precatalyst conversion.

Our results have shown that the catalyst potentially operates via various pathways depending on the acid strength, acid concentration, and solvent. We summarize these possible mechanisms in Scheme 1. The catalytic cycles initiate with the reduction of the Co(aPPy) complex as synthesized (**I**), generating a formal Co^I^ complex (**II**). In organic media with HBF_4_ as a proton source, after reduction (*E*), a protonation coupled to an electron transfer (*EC*) follows, resulting in intermediate **IV**. After protonation (*C*) of this intermediate, hydrogen is produced and **I** is regenerated. In the presence of HBF_4_, we propose that catalysis thus occurs via the *E*(*EC*)*C* mechanism (Scheme 1, blue path). When the weaker acid HNEt_3_BF_4_ is used as a proton source, intermediate **II** needs to be further reduced to provide ^0^ (**III**) before the catalytic reduction of protons takes place. Together with the pH independency of the catalytic onset potential, this points to an *EECC* mechanism in organic media (Scheme 1, green path), in agreement with the mechanistic studies of Alberto et al.^19^

Since the redox events in water changed over successive CV cycles, we propose that the as-synthesized Co(aPPy) complex is a precatalyst in this medium. This precatalyst conversion into the active species occurs via a reduction followed by a protonation at pH ≤ 5, and after conversion, a new catalytic pathway opens up (Scheme 1, right cycle). The potential of the first reduction (*E*) remains pH-independent, transforming species **V** to intermediate **VI**. After the (*EC*) step, intermediate **VII** is formed, and the catalytic cycle is closed by a final protonation (*C*), during which H_2_ is evolved, and complex (**V**) is regenerated. Although we propose that the catalyst follows a similar *E*(*EC*)*C* path at different pH values, the lifetimes of the various catalytic intermediates depend on whether the proton relay is protonated (as described in the previous section).

Our study thus shows that the earlier proposed mechanisms by the groups of Smolentsev, Ianuzzi, and Alberto (Figure 2), which might seem contradicting at first glance, could actually be closely related. We found experimental support for all four mechanisms, depending on the solvent and its acidity. In the proposed pathways by the groups of Iannuzzi and Gurdal^24^ and Alberto et al.,^19^ the pyridine ligand remains coordinated in organic solvents and was proposed to occur via an *EECC* or *ECEC* mechanism. We found support for these claims during our measurements in organic media. On the other hand, the pyridine dissociates in the proposed mechanisms of Smolentsev et al.^20^ This hypothesis is backed up by our studies in water, which substantiated the idea that the complex with the dissociated pyridine ligand is the active species.

### Influence of the Mechanism on the Catalytic Performance

Finally, to understand the implications of the different mechanisms on the catalytic performance of Co(aPPy), we carried out constant potential bulk electrolysis (CPE), using a mercury pool electrode in a custom-made H-cell (Figure SI2). We followed the current and passed charge for 1 h at pH 3.0, 4.5, and 6.0 (see Supporting Information). We also attempted to compare the performance of Co(aPPy) to that of the widely studied water-soluble Co(dmgBF_2_)_2_ cobaloxime HEC.^41^ Unfortunately, Co(dmgBF_2_)_2_ decomposed almost immediately under acidic, reductive conditions, which hampered comparison of the results (further described in Section SI10).

The results of the bulk electrolyses are provided in Table 1. We calculated the catalytic turnover from the charge that passed through the cell over the course of the electrolysis. After CPE at −1.2 V versus Ag/AgCl for 1 h, the highest catalytic TON was observed at pH 3.0 (2587), followed by pH 4.5 (1747) and pH 6.0 (355). We also investigated the stability of the catalyst by monitoring the change in catalytic current (*i*_cat_) over time, by comparing the *i*_cat_ at 30 and 60 min. The highest TON at pH 3.0 can be first explained by the relatively high catalytic activity at this pH. Under these acidic conditions, we proposed earlier that the pyridine ligand is protonated, and the substrate is present in great abundance. Together, this enables fast catalysis, also represented by the relatively high currents (Figure SI29). Second, the catalyst also showed the highest stability at this pH, indicated by the low decrease in *i*_cat_ of only 5% (Table SI6). The catalyst stability lowered at pH 4.5 and 6.0, indicated by a decrease in *i*_cat_ of 41 and 52%, respectively. We thus propose that the highest TON at pH 3.0 is a result of the (1) substrate availability, (2) high catalytic activity due to the protonated pyridine ligand, and (3) good catalytic stability.

**Table 1:** Constant Potential Electrolysis Results of Co(aPPy) at Varying pH, for 1 h at −1.2 V vs Ag/AgCl in a Divided Cell^a^

pH	TON (1 h)^b^	*i*_cat_ decrease (%)^b,c^
3.0	2587	5
4.5	1747	41
6.0	355	52

a A mercury pool electrode was used as the WE and a Pt wire as the CE, combined with a Ag/AgCl (3 M KCl) RE; (b) the TONs and *i*_cat_ were corrected for the background activity of the Hg electrode by subtraction of the charge or current measured in the absence of the catalyst. (c) Calculated from the decrease in current between 30 and 60 min.

Interestingly, these observations differ from previous reports, which stated that the catalytic stability of Co(aPPy) peaked around pH 4.5–5.^21^ In their work, Co(aPPy) was relatively unstable at pH 3.0 (TON_max_ of 901 after ±10 h), whereas a TON_max_ of 7086 was found at pH 4.5 (after 50 h). For the sake of comparison, we carried out prolonged electrolysis of Co(aPPy) at −1.2 V versus Ag/AgCl at pH 4.5 (Figure SI31). We found a TON_max_ value of 6111, which is comparable to the earlier reported value under photochemical conditions. Nevertheless, the high catalyst stability that we observed at pH 3.0 stands in stark contrast with the low TON_max_ earlier reported by Probst and co-workers. A possible explanation for this discrepancy between the reported and the herein observed stability could be the decomposition of other reagents that are present in the photochemical reaction, such as Ru(bpy)_3_^2+^.^22,25,32,42^ Comparison between the electrocatalytic and photocatalytic stabilities of the Co(aPPy) HEC should therefore proceed with caution.

**Figure 8:**
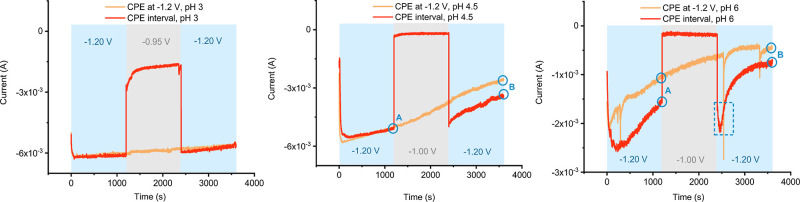
Chronoamperograms of the 60 min bulk electrolyses of [Co(aPPy)Br]Br in Milli-Q (containing 0.1 M citric acid and 0.1 M Na_2_SO_4_), at a potential of −1.2 V (orange), and of those with a 20 min interval at −1.0 V. The experiments were carried out at pH 3 (left), pH 4.5 (middle), and pH 6 (right).

Co(aPPy) showed the lowest stability at pH 6, and two main degradation pathways have been reported for Co-based HECs: (1) via an unstable Co^I^ intermediate, which can lead to disproportionation reactions or metal ion dissociation^32,43,44^ or (2) via protonation of the pyridyl ligands, ultimately resulting in dissociation from the cobalt center.^19,32,33^ In water, the latter pathway is less likely since the catalyst was most stable under strongly acidic conditions. We thus studied the influence of the Co^I^ intermediate on the overall performance via CPE, where we applied a potential of −1.2 V for 20 min, followed by a potential of −1.0 V for 20 min, during which Co(aPPy) is present as the Co^I^ species but does not catalyze proton reduction, and finally for another 20 min at −1.2 V. We compared these results to the CPE we carried out before. If the presence of the Co^I^ intermediate would be detrimental to the catalyst stability, we would expect a lowered activity in the second 20 min interval. The results of these experiments are presented in Figure 8.

Notably, at none of the pH values did we find a decreased activity after the −1.0 V interval (see also Table SI8). It is therefore unlikely that the Co^I^ intermediate initiates catalyst decomposition, as this would have led to a decreased activity in the second interval at −1.2 V. At pH 4.5 and 6.0, we even observed an increased activity after the intermission at −1.0 V. Catalyst decomposition thus occurs during catalysis, at −1.2 V, and is more pronounced at higher pH.

UV–vis titration of the Co(aPPy) complex (Figure SI32) from acidic to basic pH (3–12) revealed that the Co(aPPy) complex indeed converts irreversibly to a different species upon introduction of a strong base (5 M NaOH). A precipitate visibly formed in the cuvette with increasing pH, and a concomitant increase of the baseline was observed. Upon decreasing the pH back to 3, the original UV–vis absorption spectrum was not retrieved, even not after 4 days. We therefore looked into cobalt species that are usually only present at higher pH, by investigating the Pourbaix diagrams of analogous complexes.^13,21^ According to the Pourbaix diagram of Co(aTPy), Co^II^–OH forms at a pH higher than 9.4, and its formation was found to be partially irreversible.^13^ Another report also emphasized the strong binding (and concomitant slow exchange) of the hydroxy ligand to Co^III^(DPA-bpy).^18^ Possibly, local pH gradients could lead to the formation of such cobalt–hydroxyl complexes in the case of Co(aPPy), which could (temporarily) deactivate the catalyst. This hypothesis is supported, first, by the sudden increase in current after the −1.0 V interval at pH 6 (Figure 8, blue dashed box). During this 20 min interval, the Co–OH species might have (partially) converted back to a Co–OH_2_ complex, reactivating the catalyst. Second, the formation of Co–OH complexes as inactive intermediates could also explain the relatively high catalyst stability of Co(aPPy) at acidic conditions, where hydroxide species are unlikely to form. Finally, the proposed pyridine proton relay might also play a crucial role in the prevention of Co–OH intermediates, as the preorganized proton could quickly protonate the hydroxyl species.

## Conclusions

In this study, we electrochemically probed the mechanism of the Co(aPPy) proton reduction catalyst in various environments. Studies in organic media indicate that the catalyst operates via an *E*(*EC*)*C* pathway in the presence of a strong acid (HBF_4_) and via the *EECC* mechanism when using a weaker acid (HNEt_3_BF_4_). The acid strength also controls the catalyst stability: the complex remains stable up to 1 equivalent of strong acid, whereas the complex can tolerate up to 30 equiv of the weaker proton source.

From the studies in an aqueous environment, we propose that the Co(aPPy) complex itself is the precatalyst to an electrocatalytically active species in which one pyridine ligand has dissociated. Once this active catalyst has formed, proton reduction proceeds via an *E*(*EC*)*C* mechanism. The dissociated pyridine ring could then function as a proton relay, which potentially allows for fast protonation of the Co^I^ intermediate at low pH (≤5), resulting in high catalytic activity. In addition, we found that the catalyst is most stable in strongly acidic media, possibly due to the prevention of an inactive Co–OH species. Overall, the catalyst thus functions optimally in strongly acidic conditions, where the activity and stability is the highest.

## Supporting Information Available

Details of syntheses, characterization, electrochemistry experiments, and supporting data and figures (PDF)

## Abbreviations

aPPy: alternative penta-pyridine
*C*: chemical step
CE: counter electrode
CPE: constant potential electrolysis
CV: cyclic voltammetry
DMF: dimethylformamide
*E*: electrochemical step
EC: coupled electrochemical and chemical step
HEC: hydrogen evolution catalyst
HMDE: hanging mercury drop electrode
RE: reference electrode
TON: turnover number
WE: working electrode
