# Supplementary material for: Mechanistic Insights into Electrocatalytic Hydrogen Evolution by an Exceptionally Stable Cobalt Complex
Source: Inorg Chem. 2024 Apr 19;63(18):8484–92. doi: 10.1021/acs.inorgchem.4c01043 (PMC11080059; doi:10.1021/acs.inorgchem.4c01043)
Supplement: Supplementary file 1 — ic4c01043_si_001.pdf [file ic4c01043_si_001.pdf]

Supporting Information:

MECHANISTIC INSIGHTS INTO ELECTROCATALYTIC HYDROGEN EVOLUTION BY AN EXCEPTIONALLY STABLE COBALT COMPLEX

Maria B. Brands, Joost N. H. Reek*

University of Amsterdam, Homogeneous, Supramolecular and Bio-Inspired Catalysis, Van ‘t Hoff Institute for Molecular Sciences, Science Park 904, 1098 XH, Amsterdam, The Netherlands.

* To whom correspondence should be addressed: j.n.h.reek@uva.nl

Table of Content

1. Experimental methods S3
1.1 General considerations S3
1.2 Electrochemistry S4
1.2.1 Cyclic voltammetry in organic media S4
1.2.2 Cyclic voltammetry in aqueous media S4
1.2.3 Bulk electrolyses S4
1.3 UV-vis spectroelectrochemistry S6
1.4 Synthesis and characterization S6
1.4.1 6-bromo-2,2'-bipyridine (**1**)[11] S6
1.4.2 [2,2'-bipyridin]-6-yl(pyridine-2-yl)methanone (**2**)[10,12] S8
1.4.3 aPPy (**3**)[10] S10
1.4.4 [Co(aPPy)Br]Br (**4**)[10] S12
1.4.5 [Co(aPPy)]$(ClO_4)_2$ (**5**) S18
1.4.5 Co($dmgBF_2$) (**6**)[20] S18
2. Other Co^{II} polypyridyl complexes and their mechanisms S20
3. Acetic acid as proton source S25
4. Additional notes on HBF_4 as proton source S26
5. Screening of buffer solutions S27
6. [Co(aPPy)Br]Br compared to [Co(aPPy)(H_2O)]$(ClO_4)_2$ S29
7. The transformation of the Co(aPPy) precatalyst to a different species S30
8. Ruling out adsorption of the complex onto the Hg electrode S31
9. Mononuclear or binuclear catalysis S32
10. Constant potential electrolysis with Co(aPPy) and $Co(dmgBF_2)_2$ S33
11. UV-vis titration of the Co(aPPy) complex from acidic to basic pH S36

1. Experimental methods

1.1 General considerations

2,6-dibromopyridine, $Pd(PPh_3)_4$ and 2-tributylstannylpyridine were of commercial grade and used without further purification. $HNEt_3BF_4$ was synthesized according to literature procedure.[1] CD_2Cl_2 was purchased from Eurisotop. Spectroscopic grade MeOH was obtained from Biosolve. Milli-Q water (18.2 MΩ·cm^{-1}) was used for all aqueous electrochemical studies and was obtained from a Milli-Q IQ 7000 water purification system (Merck). Dry MeOH was obtained via distillation from CaH_2 under N_2 atmosphere. Dry Et_2O and toluene were obtained from an MBraun solvent purification system and stored on 3 Å molecular sieves (molsieves) under Ar atmosphere. Dimethylformamide (DMF) and ethyl picolinate were of commercial grade and dried over 3 Å molsieves under Ar atmosphere for at least 24 hours prior to use. An automated flash column chromatography system (Büchi model Pure C-810 Flash), equipped with a solid loader for dry product loading (Büchi product 11068975), was used for purification.

^{1}H nuclear magnetic resonance (NMR) spectra were recorded on a Bruker DRX 500, Bruker AMX 400 or Bruker DMX 300 spectrometer at room temperature. The reported ppm values are relative to $SiMe_4$ by referencing the residual solvent peak to $SiMe_4$.[2]

Mass spectra (MS) were collected on an Advion Expression L Compact Mass Spectrometer (Advion Inc., USA). Samples were analyzed by flow injection analysis. For electrospray ionization (ESI), typical measurement conditions are as follows. Positive ion-mode: ESI voltage 3500 V, source gas temperature 200 °C, capillary temperature 250 °C. Flow injection with a flow rate of 0.1 mL/min. All mass spectra were recorded with an average duration of 1 min.

High-resolution mass spectra (HR-MS) were collected on an AccuTOF LC, JMS-T100LP Mass spectrometer (JEOL, Japan). For ESI, typical measurement conditions are as follows: positive ion-mode, needle voltage 2500 V, Orifice 1 voltage 120 V, Orifice 2 voltage 9 V, ring lens voltage 22 V, Orifice 1 80 °C, desolvating chamber 250 °C; flow injection with a flow rate of 0.01 mL/min. All mass spectra were recorded with an average duration of 1 min.

EPR measurements were performed in air-tight J-Young quartz tubes in an atmosphere of purified nitrogen. EPR spectra were recorded on a Bruker EMX-plus CW X-band spectrometer equipped with a Bruker ER 4112HV-CF100 helium cryostat. The spectra were obtained on freshly prepared solutions of 1–10 mM [Co(aPPy)Br]Br and simulated using EasySpin[3] via the cwEPR[4] GUI. EasySpin, a comprehensive software package for spectral simulation and analysis in EPR.

Single crystals of [Co(aPPy)Br]Br and [Co(aPPy)]$(ClO_4)_2$ suitable for X-ray diffraction were measured with a Bruker D8 Quest Eco diffractometer equipped with a Triumph monochromator (λ = 0.71073 Å) and a CMOS Photon 100 detector at a temperature of 150 ± 2 K. Intensity data were integrated with the Bruker APEX2 software.[5] Absorption correction and scaling was performed with SADABS.[6] The structures were solved using intrinsic phasing with the program SHELXT.[7] Least-squares refinement was performed with SHELXL-2013[8] against F^2 of all reflections. Non-hydrogen atoms were refined with anisotropic displacement parameters. The H-atoms were placed at calculated positions using the instructions AFIX 13, AFIX 43 or AFIX 137 with isotropic displacement parameters having values 1.2 or 1.5 times U_{eq} of the attached C atoms.

UV-vis measurements (both steady state as well as spectroelectrochemical) were conducted on a Hewlett-Packard 8453 spectrometer.

1.2 Electrochemistry

1.2.1 Cyclic voltammetry in organic media

Cyclic voltammograms in organic media were recorded using a PGSTAT101 potentiostat (Autolab). The measurements were performed in a heart-shaped cell under Ar atmosphere in 1 mM solutions of analyte in dry DMF (degassed by sparging with Ar for 10 min), containing 0.1 M $n\mathrm{Bu_4NBF_4}$ as the supporting electrolyte. The cell was equipped with a glassy carbon working electrode (WE; Tianjin Aida Hengsheng Technology Development Co., Ltd., 3 mm diameter), a leakless Ag/AgCl (3 M KCl) reference electrode (RE; eDAQ, ET069) and a Pt wire (diameter 0.5 mm) counter electrode (CE). The glassy carbon electrode was polished using aluminum oxide powder (grain size 0.3 µm, Metrohm), by moving it over a polishing cloth (Metrohm) in a "∞" movement, for eight times, after which the cloth was turned 90°. These steps were then repeated another seven times. At the end of each measurement series, ferrocene was added to the electrolyte solution, and a $E_{1/2}$ ($Fc^{+/0}$) between +0.495 and +0.505 V versus Ag/AgCl was measured in DMF. All the measurements were carried out at room temperature.

1.2.2 Cyclic voltammetry in aqueous media

Cyclic voltammograms were recorded using a 663 VA stand with an IME663 interface (Autolab), in combination with a PGSTAT101 potentiostat (Autolab). As working electrode, a static hanging mercury drop electrode (HMDE; drop size 2; N_2 pressure of 1.5 bar) was used, a glassy carbon rod (Metrohm product 6.1241.020 and 6.1247.000) as auxiliary electrode, and a Ag/AgCl (3 M KCl) electrode as reference electrode (Metrohm products 6.0728.120 and 6.1245.010).

The analyte solution consisted of 1 mM analyte (unless stated otherwise), 0.1 M $NaBF_4$ as supporting electrolyte, and 0.2 M citric acid buffer, adjusted to the pH of interest using 5 M NaOH (99.99% pure), dissolved in Milli-Q. Prior to the measurement, the cell was degassed by bubbling Ar through for at least ten minutes, and since the setup was not air-tight, the measurements were carried out under a gentle Ar flow. The measurements were carried out at room temperature.

The CV measurements were performed as automated sequences to maximize reproducibility. After each voltammogram, the implemented stirrer was automatically turned on for 10 s. Subsequently, a command was inserted to push through five new drops of mercury through the capillary, to avoid decomposition artefacts on subsequent measurements. For the scan rate-dependent studies, twelve voltammograms were recorded in each sequence, varying the scan rate as follows: 30, 25, 20, 15, 10, 5, 2.5, 1, 0.5, 0.25 and 0.1 $V{\cdot}s^{-1}$. The potential window was varied from 0 V to –2, –1.9, –1.8, –1.7, –1.6, –1.5, –1.4, –1.3, –1.2, –1.1 or –1.0 V.

1.2.3 Bulk electrolyses

Bulk electrolysis in an undivided cell (see Figure SI1) was carried out using the 663 VA stand with an IME663 interface (Autolab), in combination with a PGSTAT101 potentiostat (Autolab). As working electrode, a continuously dropping mercury electrode (DME; N_2 pressure of 1.5 bar) was used, a glassy carbon rod (Metrohm product 6.1241.020 and 6.1247.000) as auxiliary electrode, and a Ag/AgCl (3 M KCl) electrode as reference electrode (Metrohm products 6.0728.120 and 6.1245.010). Bulk electrolyses were carried out at

potentiostatic conditions at room temperature (see Section SI9 for more details), while stirring the reaction mixture with the integrated stirrer, set at 3000 rpm.

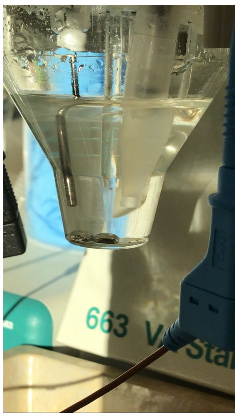

Figure SI1. A picture of the undivided bulk electrolysis cell.

Bulk electrolysis in a divided cell (Section SI10) was carried out using a custom-made H-cell, as depicted in Figure SI2. A glass frit (3 mm) with a P3 porosity separated the anodic and cathodic compartment. A mercury pool was used as working electrode (d = 2.2 cm), which was connected to the potentiostat (PGSTAT101 or PGSTAT302N, Autolab) using a Pt wire that was melted through the bottom of the glass. Pt wire (d = 0.5 mm) was used as counter electrode, and a Ag/AgCl in 3 M KCl reference electrode (eDAQ, ET069) was positioned 2 cm away from the Hg pool. The distance between the working and counter electrode was approximately 3.2–5.5 cm. The same anolyte and catholyte was used, based on 0.1 M Na_2SO_4 and 0.1 M citric acid in milli-Q. The pH was increased to the desired value using 5 M NaOH (99.99% pure), after which the catalyst (Co(aPPy) or $Co(dmgBF_2)_2$) was added, resulting in a catalyst concentration of 5 μM. The total volume of the catholyte was 8 mL, and of the anolyte 12 mL. Prior to electrolysis, the anolyte and catholyte were simultaneously purged with Ar for at least 10 min.

Bulk electrolysis was carried out under potentiostatic conditions (see section SI10 for more details), at room temperature, while stirring the catholyte at 600 rpm with a small stirring bean (1 cm in length), that floated on the Hg pool. We did not compensate for the IR drop.

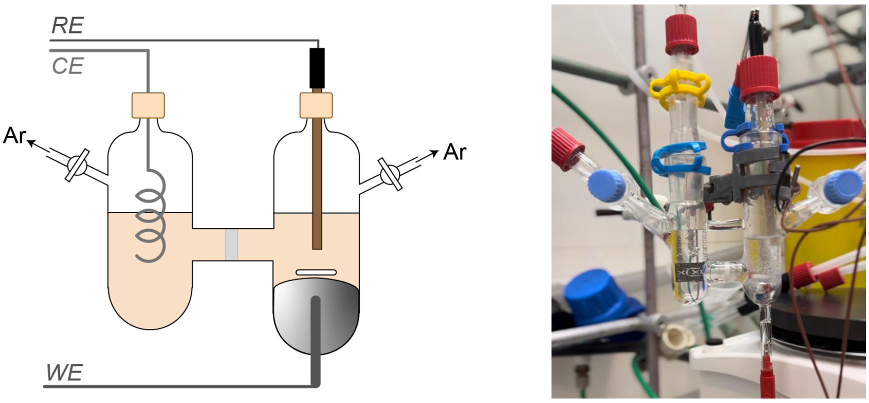

Figure SI2. Bulk electrolysis setup using a mercury pool electrode in a divided H-cell. On the left, a schematic overview is shown, and on the right a picture of this setup is displayed.

1.3 UV-vis spectroelectrochemistry

UV-vis spectroelectrochemical measurements were performed with an optically transparent thin-layer electrochemical (OTTLE) cell equipped with CaF_2 optical windows and a micro-grid platinum working electrode.[9] The cell was connected to a PGSTAT101 potentiostat (Autolab) and a Hewlett-Packard 8543 UV-vis spectrometer. A scan rate of 3.75 mV/s was applied, to allow for semi-quantitative electrochemical conversion, while measuring the UV-vis spectrum every 20 seconds. Measurements were carried out at room temperature.

1.4 Synthesis and characterization

The synthesis route of the [Co(aPPy)Br]Br complex was based on the already reported procedure by Alberto et al.,[10] and additional characterization was carried out. The main differences can be found in the scale and in the purification methods. A simplified synthesis overview can be found in Scheme SI1, followed by a detailed procedure of the steps.

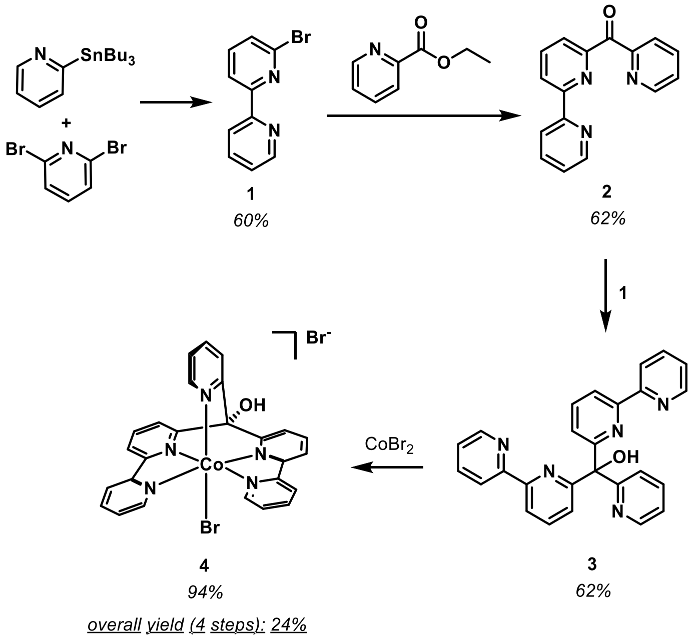

Scheme SI1. Overview of [Co(aPPy)Br]Br synthesis (**4**)

*1.4.1 6-bromo-2,2'-bipyridine (**1**)*[11]

Due to the large scale of this reaction, rather concentrated acid and base solutions are used in the acid-base extraction. To prevent undesired events, care should be taken to slowly add the two to each other.

To a flame-dried three-neck 3 L round-bottom flask, 2,6-dibromopyridine (57.0 g, 241 mmol, 1.03 equiv.) and $Pd(PPh_3)_4$ (13.5 g, 11.7 mmol, 5 mol%) were added and left under vacuum for 30 min. Toluene was added (dry, 1 L) via a cannula, resulting in a brown turbid solution. 2-(tributyltin)pyridine was added with a syringe and needle (two portions of 38.0 mL, 233 mmol, 1 equiv.). The solution was refluxed under argon atmosphere at 110°C overnight.

Toluene was evaporated, after which 250 mL DCM and 500 mL 6 M HCl were added to the concentrate, resulting in a two-layer system. The two layers were separated and the organic layer was washed with 6 M

HCl (500 mL, twice). Two setups were build consisting of a 500 mL dropping funnel and a 3 L three-neck round-bottom containing a 5 × 2.5 cm oval stirring bar, which was put in a salt/ice bath of adequate size. The round-bottoms were filled with ammonia solution (25% w/w, 700 mL, twice) and the dropping funnels were filled with the aqueous layer. The aqueous layer was added dropwise to the ammonia solution over the course of at least two hours, leading to the formation of a white precipitate. Care was taken that the mixture remained properly stirred, although at a certain point the amount of precipitate blocked the stirring bar completely. DCM (200 mL, twice) was added to dissolve the white precipitate and restart the stirring.

The aqueous layers were extracted with DCM (800 mL, twice) and the organic layer was washed with ammonia solution (10%, 250 mL, twice) and water (200 mL, twice). The solution was dried over Na_2SO_4, after which the solvent was evaporated. A dark brown oil remained that crystallized over time. The brown solid was dissolved in EtOAc and filtered over silica, after which the product was impregnated on 13.3 g of Celite. The Celite-impregnated powder was purified using an auto-column equipped with a solid loader and an 80 g silica column (Büchi product 140000025). The detailed method is displayed in Table SI1.

Table SI1. Separation method for the purification of **1**.

Time (min)	Heptane (%)	EtOAc (%)	Flow rate (mL·min^{-1})
0	100 → 99	0	30
2			60
5	99 → 98	1	60
10	98 → 97	2	60
15	97 → 95	3	60
20	95 → 92	5	60
25	92 → 90	8	60
35	90	10	60
45	90 → 0	10 → 100	60
48	0	100	60
53	0 → 100	100 → 0	60
55	end	end	60

The Celite mixture was divided over ten columns, charging the solid loader with approximately 8 g per run. Care should be taken to keep hand-tightening the solid loader during the column, as it becomes looser when more and more product dissolves from the Celite into the column, which can cause leaks. After combining all the pure fractions (analyzed by thin-layer chromatography analysis), the product was obtained as a white crystalline powder (34.1 g, 145 mmol, 60%).

^{1}H NMR (400 MHz, $CDCl_3$, δ): 8.67 (ddd, J = 4.8, 1.8, 0.9 Hz, 1H), 8.41 (t, J = 8.2 Hz, 2H), 7.83 (td, J = 7.8, 1.8 Hz, 1H), 7.68 (t, J = 7.8 Hz, 1H,), 7.50 (dd, J = 7.8, 0.9 Hz, 1H), 7.34 (ddd, J = 7.4, 4.8, 1.2 Hz, 1H).

MS–ESI (*m/z*): $[M + H]^+$ calcd for $C_{10}H_8BrN_2$, 235.0; found, 235.1.

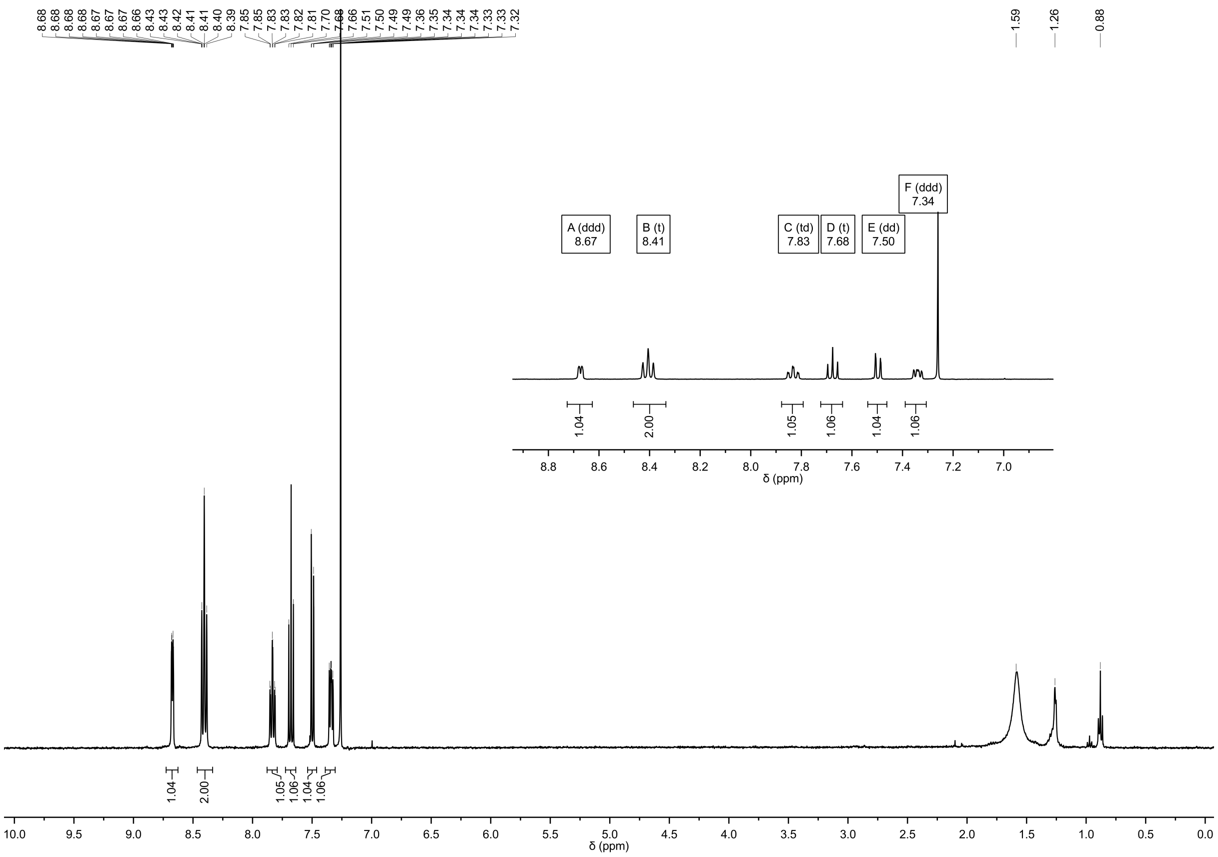

Figure SI3. ^{1}H NMR spectrum of **1**, the inset shows a zoom-in on the aromatic region.

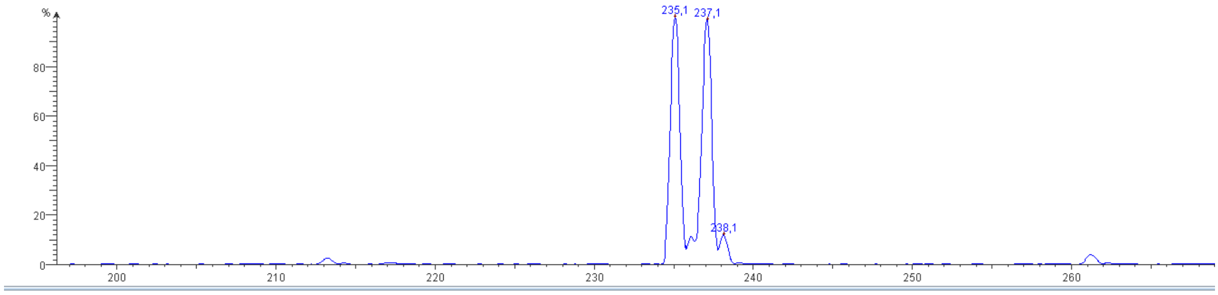

Figure SI4. ESI mass spectrum of **1**.

*1.4.2 [2,2'-bipyridin]-6-yl(pyridine-2-yl)methanone (**2**)*[10,12]

1 (16.0 g, 68.1 mmol, 1 equiv.) was dissolved in Et_2O (dry, 300 mL) in a flame-dried Schlenk flask, and cooled to –78 °C over the course of 30 min, resulting in a white suspension. *n*-BuLi (2.5 M in hexanes, 30 mL, 75 mmol, 1.1 equiv.) was added dropwise over the course of 45 min, resulting in a dark red solution. The reaction was stirred at this temperature for 1 h, after which ethyl picolinate (dry, 10.5 mL, 77.1 mmol, 1.1 equiv.) was added over the course of 15 min. Directly after the addition, the –78 °C bath was replaced with a –40 °C ethylene glycol/ethanol/salt bath. The reaction was stirred at this temperature for 1 h, during which a change to a blue-green suspension was observed. The –40 °C bath was exchanged for a 0 °C ice bath and stirred at this temperature for 1 h, after which the mixture was cooled to –78 °C again. Dry MeOH (30 mL) was added over the course of 30 min to quench the reaction, resulting in a dark red solution. DCM was added and the Schlenk was exposed to air, after which 2 M HCl was added and the product was extracted into the aqueous layer. The extraction with 2 M HCl was repeated until the aqueous layer remained colorless during washing.

The aqueous layer was basified with 5 M NaOH carefully until pH 14, and extracted with DCM until the organic layer became colorless. The organic layer was then dried over Na_2SO_4, filtered, and the solvent was evaporated. The remaining brown oil was redissolved in DCM and impregnated onto Celite, using enough Celite until a loose powder (brown) was obtained (total mass crude product and Celite: 34.6 g). The product was purified with an auto-column equipped with a solid loader and a 25 g silica column (Büchi product 140000023). The detailed method is displayed in Table SI2. The Celite mixture was divided over eight columns, charging the solid loader with approximately 6.5 g per run. After combining all the pure fractions (analyzed by mass spectrometry), the product was obtained as a brown crystalline powder (11.1 g, 42.5 mmol, 62%).

Table SI2. Separation method for the purification of **2**.

Time (min)	Dichloromethane (%)	Acetone (%)	Flow rate (mL·min^{-1})
0	100	0	32
15	100 → 98	0 → 2	32
19	98 → 96	2 → 4	32
23	96 → 94	4 → 6	32
27	94 → 90	6 → 10	32
31	90 → 85	10 → 15	32
34	85 → 80	15 → 20	32
36	80	20	32
66	end	end	32

^{1}H NMR (300 MHz, CD_2Cl_2, δ): 8.74 (ddd, *J* = 4.8, 1.8, 1.0 Hz, 1H), 8.72–8.59 (m, 2H), 8.26 (dt, *J* = 8.0, 1.1 Hz, 1H), 8.10 (dt, *J* = 7.8, 1.2 Hz, 1H), 8.10–7.97 (m, 2H), 7.93 (td, *J* = 7.7, 1.7 Hz, 1H), 7.77 (td, *J* = 7.8, 1.8 Hz, 1H), 7.54 (dd, *J* = 4.8, 1.3 Hz, 1H), 7.51 (dd, *J* = 44.8, 1.3 Hz, 1H), 7.34 (dd, *J* = 4.8, 1.2 Hz, 1H), 7.32 (dd, *J* = 4.8, 1.2 Hz, 1H).

MS–ESI (*m/z*): $[M + H]^+$ calcd for $C_{16}H_{12}N_3O$, 262.1; found, 262.0.

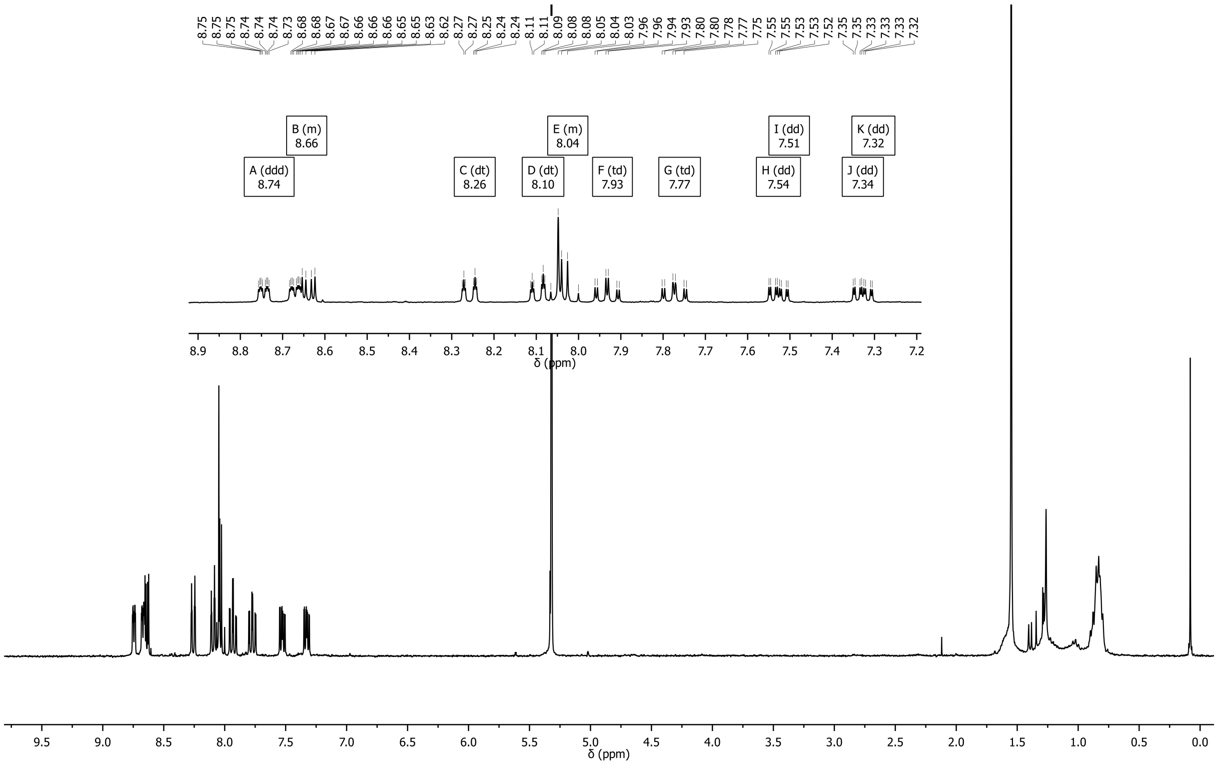

Figure SI5. ^{1}H NMR spectrum of **2**, the inset shows a zoom-in on the aromatic region.

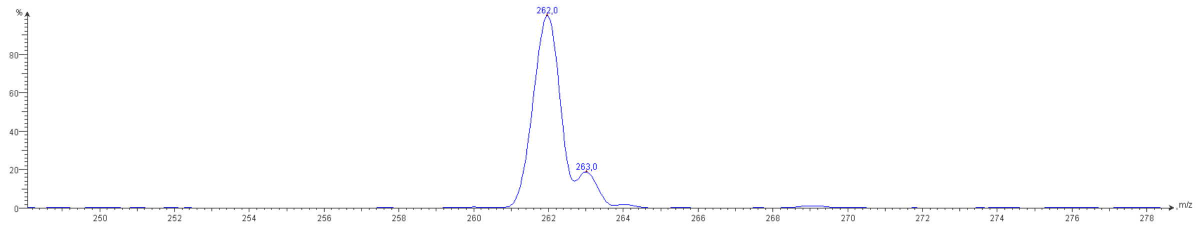

Figure SI6. ESI mass spectrum of **2**.

*1.4.3 aPPy (**3**)*[10]

1 (11.3 g, 48.0 mmol, 1.26 equiv.) was dried overnight in a flame-dried 1 L three-neck round-bottom flask under vacuum. **2** (9.97 g, 38.2 mmol, 1 equiv.) was dried in a vacuum oven overnight at 60 °C, after which the material was ground to a fine powder with mortar and pestle. The dry **1** was put under Ar and dissolved in Et_2O (dry, 500 mL), which resulted in a colorless solution. After cooling the solution to –78 °C, a white suspension was obtained. *n*-BuLi (2.5 M in hexanes, 20 mL, 50 mmol, 1.2 equiv.) was added at –78 °C over the course of 30 min, during which the suspension turned into a deep red solution. The solution was stirred at –78 °C for 1 h, after which **2** was added against an Ar flow, resulting in a color change from red to blue-green. The reaction mixture was subsequently warmed to room temperature and stirred for 1 h, during which a color shift from blue-green to deep red was observed. The reaction mixture was cooled to –78 °C and MeOH (distilled, 20 mL) was added dropwise to quench the reaction, which resulted in the formation of white fumes. The reaction mixture was warmed to room temperature overnight, which was accompanied by a change in appearance from a deep red solution to a grey-blue suspension to a brown suspension. DCM and 2 M HCl were slowly added to the reaction mixture and the product was extracted into the aqueous layer until the

aqueous layer remained colorless. The aqueous layer was basified to pH 14 with 5 M NaOH, which led to precipitation of the product. The product was subsequently extracted in DCM until the organic layer remained colorless. The organic layer was then dried over Na_2SO_4, filtered, and the solvent was removed at reduced pressure. The product was loaded on Celite and purified using an auto-column, equipped with a solid loader and a 67-gram or a 330-gram reverse phase C_{18}-silica column (Screening Devices, products custom-made). The separation method is displayed in Table SI3.

The Celite mixture was divided over ten columns, charging the solid loader with a maximum of 10.8 g per run. After combining the pure fractions (based on mass spectrometry), the product was obtained as a light-brown powder (9.91 g, 23.7 mmol, 62%).

Table SI3. Separation method for the purification of **3**.

Time (min)	MeOH (%)	1:1 H_2O/MeOH (%) [a)]	Flow rate (mL/min)
0	0	100	10
2	0	100	15
4	0	100	20
5	0	100	25
7	0	100	30
15.5	0 → 20	100 → 80	30
23			35
25.5	20	80	35
40	20	80	40
48	20	80	35
65.5	20 → 40	80 → 60	35
73			40
75.5	40	60	40
107	40 → 60	60 → 40	40
116			50
117	60	40	50
119	60	40	55
122	60	40	60
126	60	40	65
129	60	40	70
162	60 → 40	40 → 60	70
170	40	60	70
174	40 → 20	60 → 80	70
182	20	80	70
188	20 → 0	80 → 100	50
196	0	100	50
206	end	end	0

a) Due to the exothermicity of mixing water and methanol, a premixed solution was made, to prevent cracking of the column. b) flow rate was lowered due to high pressure on the column (>75 psi).

^{1}H NMR (300 MHz, MeOD, δ): 8.61 (dd, *J* = 4.8, 1.5 Hz, 2H), 8.52 (d, *J* = 4.9 Hz, 1H), 8.25 (dd, *J* = 7.8, 1.0 Hz, 2H), 8.19 (dt, *J* = 8.0, 1.1 Hz, 2H), 7.93 (t, *J* = 7.8 Hz, 2H), 7.87–7.64 (m, 6H), 7.48–7.23 (m, 3H).

MS–ESI (*m/z*): [M + H]$^+$ calcd for $C_{26}H_{20}N_5O$, 418.2; found, 418.1.

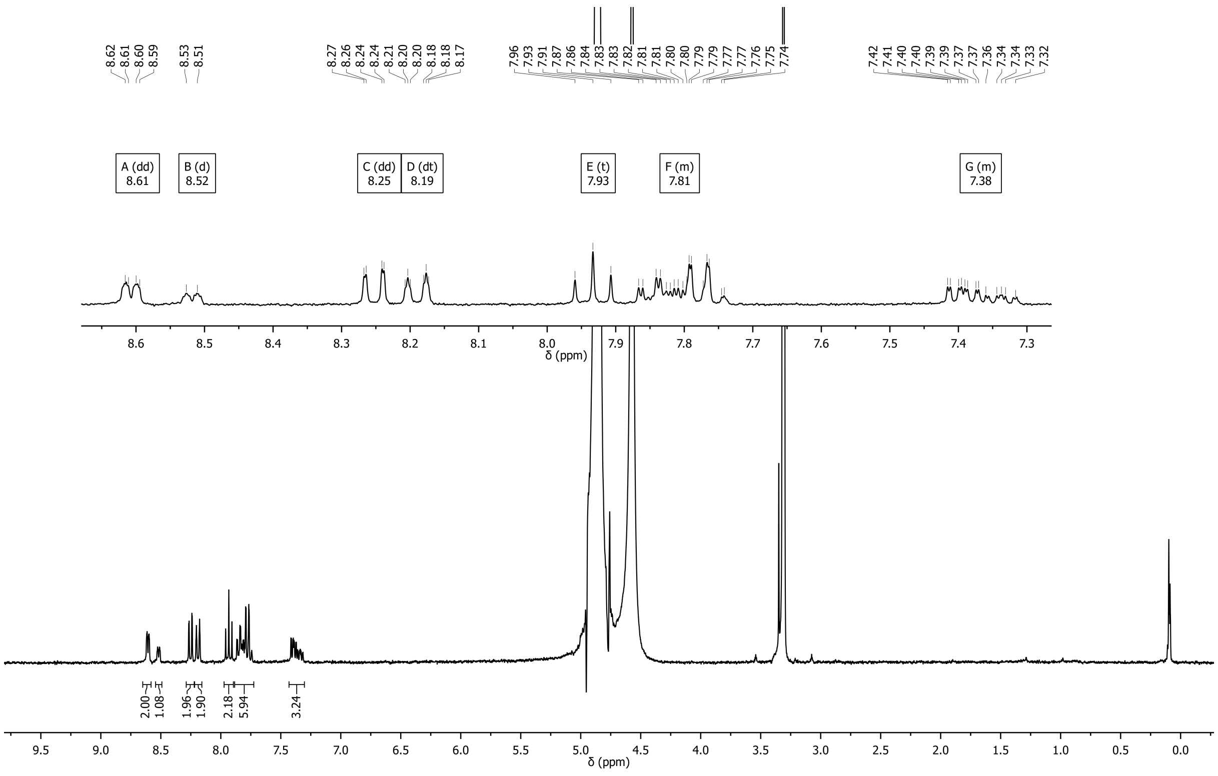

Figure SI7. ^{1}H NMR spectrum of **3**, the inset shows a zoom-in on the aromatic region.

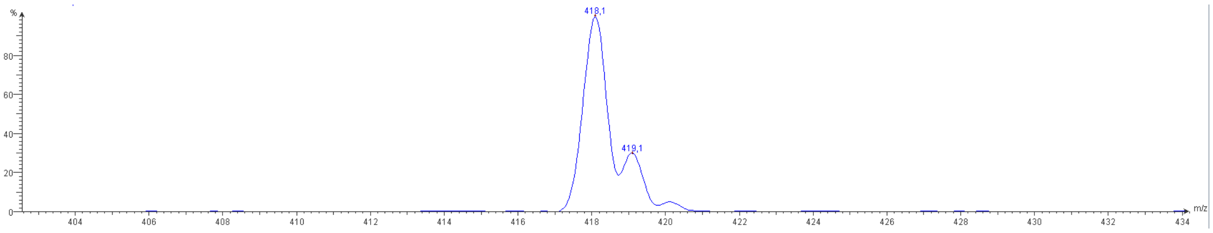

Figure SI8. ESI mass spectrum of **3**.

*1.4.4 [Co(aPPy)Br]Br (**4**)*[10]

$CoBr_2$ (10.3 mg, 47.1 µmol, 1.01 equiv.) and **3** (19.5 mg, 46.7 µmol, 1 equiv.) were dissolved in MeOH (4 mL, HPLC grade), which resulted in a brown solution that was left to stir overnight at room temperature. The resulting orange solution was filtered over Celite and the solvent was evaporated at reduced pressure. The brown film was redissolved in MeOH until a saturated solution was obtained, after which the product was crystallized out of solution via vapor diffusion with Et_2O. The blue supernatant was decanted off the crystals and the dark brown crystals were washed with Et_2O (27.9 mg, 43.8 µmol, 94%).

*This reaction was also carried out at larger scales, which led to a decrease in yield (63% or 73% for 0.48 or 0.22 mmol **3**, respectively.*

^1H NMR (300 MHz, MeOD, δ): 209.24 (br s), 79.91 (s), 67.44 (s), 63.21 (s), 59.74 (s), 48.02 (s), 26.88 (s), 18.23 (br s), 13.00 (s), 10.87 (s), 10.23 (s).

HR-MS ESI (*m/z*): $[M - Br]^+$ calcd. for $C_{26}H_{19}BrCoN_5O$, 555.0105; found, 555.0092.

UV-vis (H_2O) λ_{max} (ε): 307 (shoulder – 21581), 296 (26894), 246 (28993).

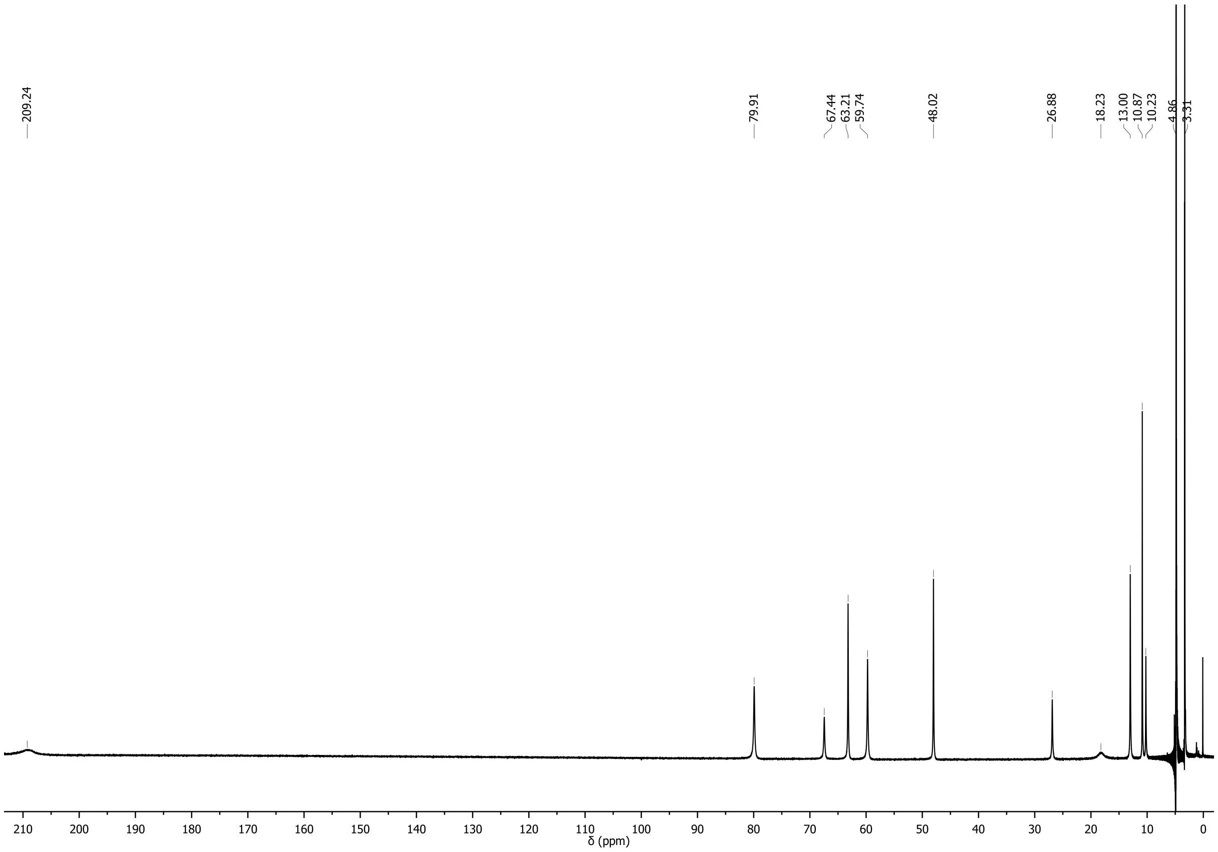

Figure SI9. ^1H NMR spectrum of **4**.

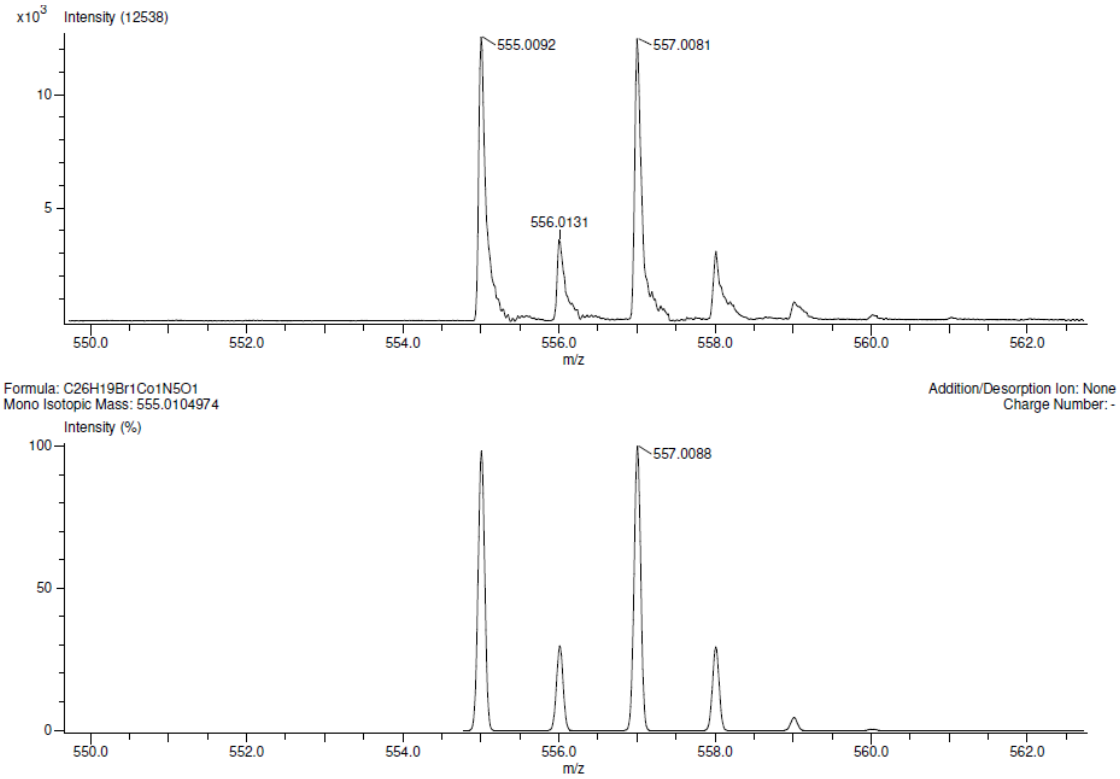

Figure SI10. HRMS spectra of **4**, where the top spectrum shows the measured data, and the bottom shows the simulated spectrum. .

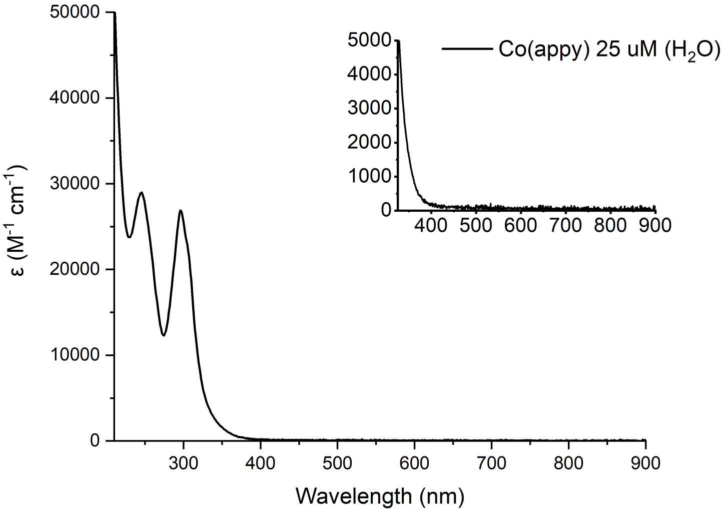

Figure SI11. UV-vis absorption spectrum of [Co(aPPy)Br]Br (25 μM) in H_2O. Inset: zoom-in on longer wavelengths.

Crystallographic details: XRD quality single crystals were grown by vapor diffusion of Et_2O into a concentrated MeOH solution of [Co(aPPy)Br]Br at room temperature. The 50% thermal ellipsoid probability plot is depicted in Figure SI12. The unit cell parameters were found to be in excellent agreement with those previously reported (Table SI4).

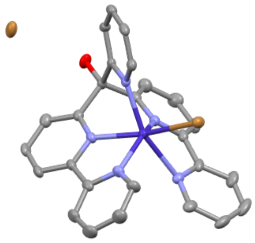

Figure SI12. 50% thermal ellipsoid probability plot of the crystal structure of **4**.

Table SI4. Comparison of unit cell parameters of the reported [Co(aPPy)Br]Br crystals and those synthesized in this work.

Parameter	Reference 10	This work
a (Å)	12.4832	12.4580
b (Å)	12.6716	12.6746
c (Å)	17.8641	17.8969
α (°)	78.7144	78.6060
β (°)	72.7054	72.4410
γ (°)	71.3303	71.3580
Volume	2540.22	2536.86
crystal	triclinic	triclinic
symmetry space group	P-1	P-1
Z	2	2

For EPR measurements, [Co(aPPy)Br]Br was dissolved in a Schlenk under N_2 atmosphere in a 1:1 mixture of H_2O and EtOH, and degassed for 15 minutes by purging the solution with nitrogen. This solution was then filtered into a J. Young valve EPR tube under nitrogen atmosphere and frozen in liquid nitrogen. The frozen sample was then brought into the EPR spectrometer and the following spectrum (Figure SI13) was measured at a temperature of 8K (MW freq. = 9.6428, MW power = 0.6325 mW, Mod. amp. = 0.4 G).

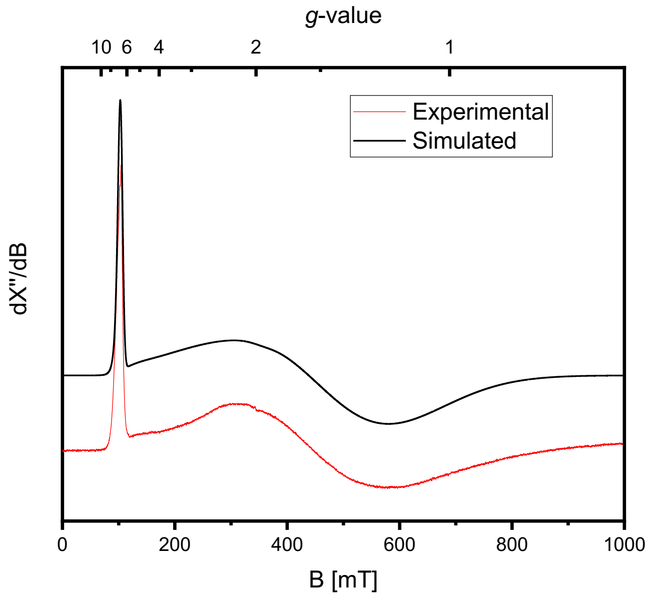

Figure SI13. Experimental (red) and simulated (black) EPR spectra of complex **4** in H_2O/EtOH 1:1 solution at 8 K.

Two features can be observed in the experimental spectrum, a sharp feature at g_{eff} = 6.67 corresponding to the |±1/2⟩ Kramers doublet and a broader feature centered around g_{eff} = 1.51 corresponding to the |±3/2⟩ Kramers doublet. Simulation of this spectrum, using EasySpin via the cwEPR gui, provided a satisfactory fit to a S = 3/2 spin system with a axial zero-field splitting parameter D = 13 cm^{-1}, and a high rhombicity of E/D = 0.23. The axial zero-field splitting parameter for [Co(aPPy)Br]Br is lower than six-coordinate N_6Co(II) complexes[13] and higher than five-coordinate N4XCo(II) complexes,[14] in accordance with the structure of this complex.

Spectroelectrochemical studies were carried out to gain more insight into the properties of the reduced species. The absorption of the Co^{II} complex shows two maxima at 247 and 297 nm, with a shoulder at 310 nm (Figure SI11). Similarly to the previously reported Co(aTPy) complex,[15] we propose that these absorptions arise from π–π* transitions of the aPPy ligand. When Co^{II} is reduced to Co^{I}, a decrease in absorption at λ_{max} of 247, 297 and 310 nm was observed (at –1.0 V vs Ag wire, see Figure SI14 and SI15). A weakly absorbing peak at λ_{max} of 295 nm remains visible. This weak absorption is unusual for Co^{I}-polypyridyl complexes, which usually show an increased absorption in the visible region compared to their Co^{II} analogues.[15–18] However, the Co(aPPy) complex was suggested to have a substantially different electronic structure than other Co^{I}-polypyridyl complexes,[10,19] which could explain this atypical behavior. Upon further decrease of the potential (up to –1.7 V vs Ag wire), broad and intense absorption features in the visible region (459 and 535 nm) arise.

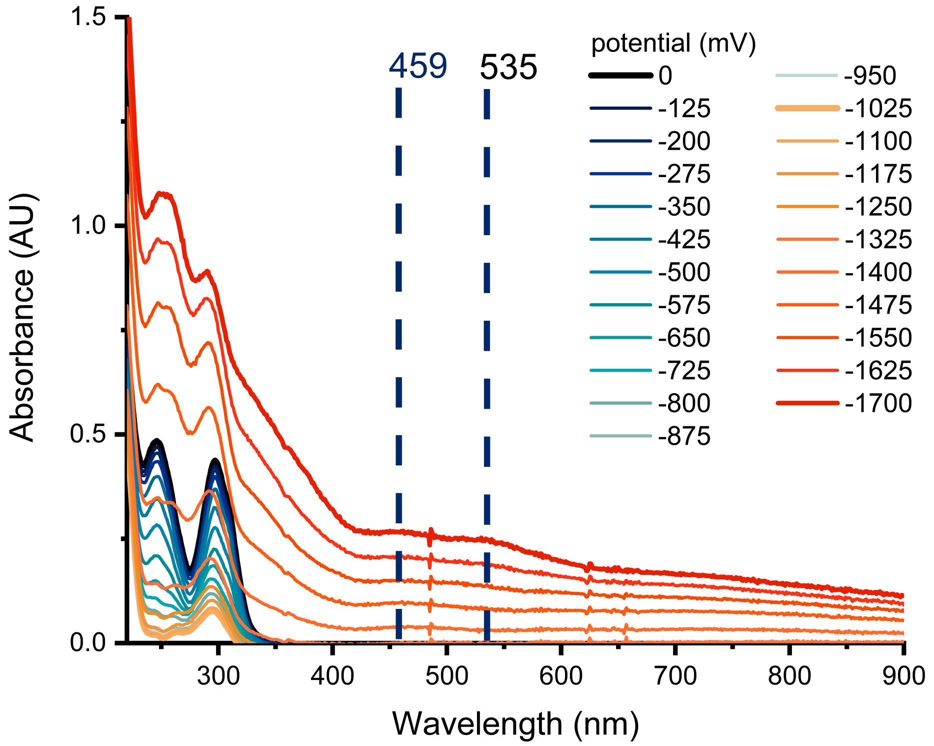

Figure SI14. UV-vis spectral evolution of 1 mM **4** in dry MeCN (containing 0.1 M $TBAPF_6$) at increasingly negative applied bias (V vs Ag wire), measured in an OTTLE cell.

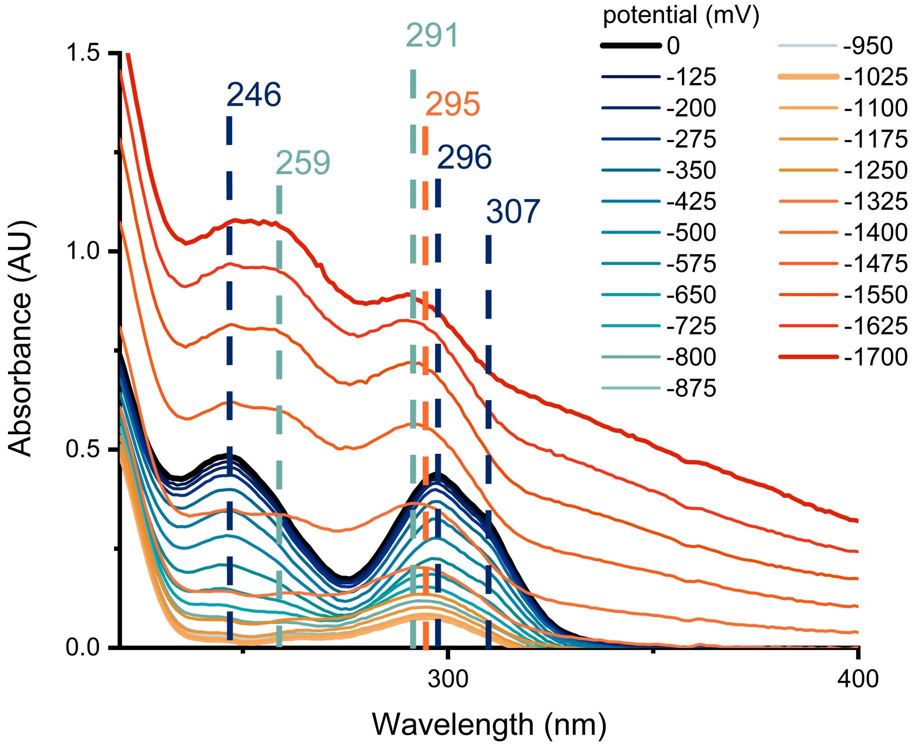

Figure SI15. Zoom in on short-wavelength spectral evolution of 1 mM **4** in dry MeCN (containing 0.1 M $TBAPF_6$) at increasingly negative applied bias (V vs Ag wire), measured in an OTTLE cell.

*1.4.5 [Co(aPPy)](ClO$_4$)$_2$ (**5**)*

Perchlorate salts of organometallic complexes are potentially explosive. Although we encountered no such problems, only small quantities should be prepared, and handled with care (e.g., by using plastic spatulas instead of metallic ones).

3 (101 mg, 242 μmol, 1 equiv.) was dissolved in a mixture of MeOH (7.5 mL, spectroscopic grade) and Et_2O (2.5 mL), after which $Co(ClO_4)_2·6H_2O$ (89.7 mg, 245 μmol, 1 equiv.) was added. The resulting solution was stirred overnight and turned into a brown solution. After 16 h, Et_2O was added and a suspension formed, which was filtered over a disposable frit filter (10 μm pore size, Screening Devices BV, product EF.075.16.10). The resulting brown residue was collected by redissolving in MeOH, which was subsequently evaporated, yielding a brown film. The brown film was recrystallized via the vapor diffusion method, using MeOH as solvent and Et_2O as antisolvent, which yielded dark brown crystals (89.6 mg, 133 μmol, 54%). The crystals were characterized by mass spectroscopy and cyclic voltammetry (in Section SI6).

HRMS–ESI (*m/z*): $[M – ClO_4]^+$ calcd for $C_{26}H_{19}ClCoN_5O_5$, 575.0401; found, 575.0393.

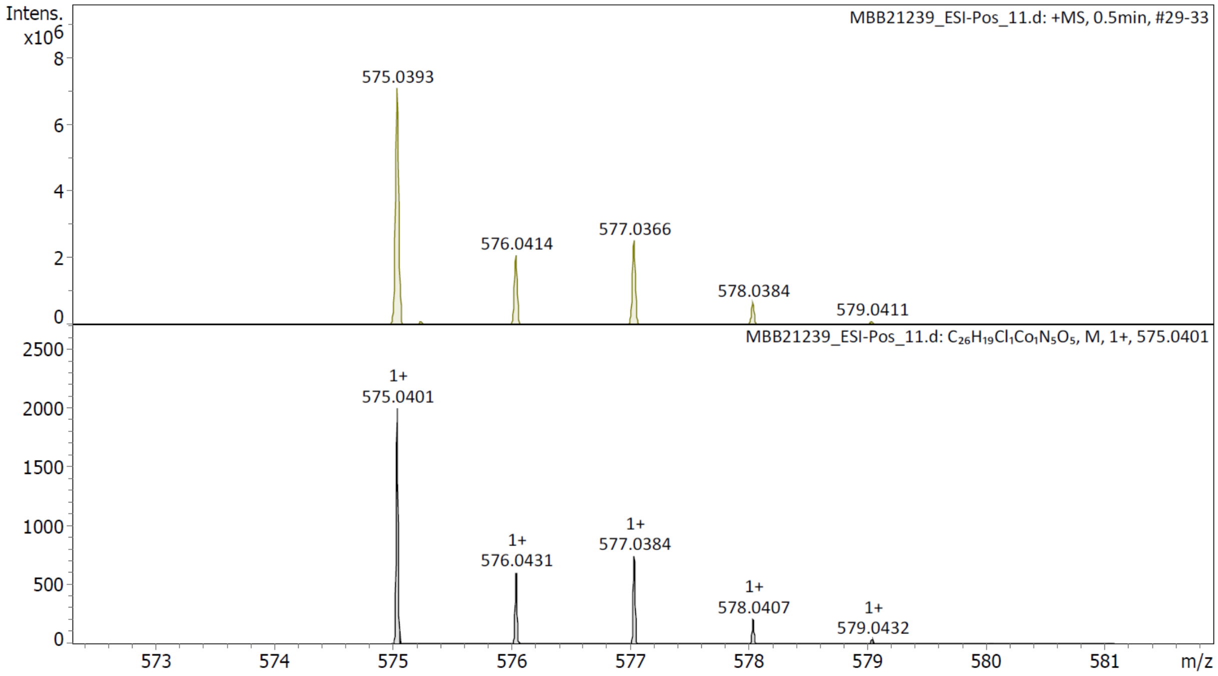

Figure SI16. HR-MS ESI spectra of **5**: at the top, the measured spectrum is shown, and at the bottom the simulated spectrum is given.

*1.4.5 Co(dmgBF$_2$) (**6**)*[20]

Suspended dimethylglyoxime (1.90 g, 16.4 mmol, 2.02 equiv.) and $Co(OAc)_2·4H_2O$ (2.01 g, 8.07 mmol, 1 equiv.) in Et_2O (150 mL), which resulted in a pink suspension. $BF_3·OEt_2$ (10 mL, 81 mmol, 10 equiv.) was added, resulting in a dark brown solution with a yellow-gold powder suspended in it. The reaction mixture was stirred under Ar atmosphere for 16 h, which resulted in a brown solution containing a fine pink solid. The reaction mixture was filtered and washed with Et_2O until the filtrate became colorless. Next, the residue was washed with ice-water, until the dark brown filtrate that was coming off the residue turned light yellow. The resulting pink powder was further purified in batches of 30 mg via crystallization from MeOH/Et_2O.

Crystallization on a larger scale was unsuccessful and led to decomposition of the product, as indicated by the product turning from a pink powder to a brown, insoluble solid.

HRMS–ESI (*m/z*): $[M]^+$ calcd for $C_{10}H_{18}B_2CoF_4N_5O_6$, 461.0711; found, 461.0728.

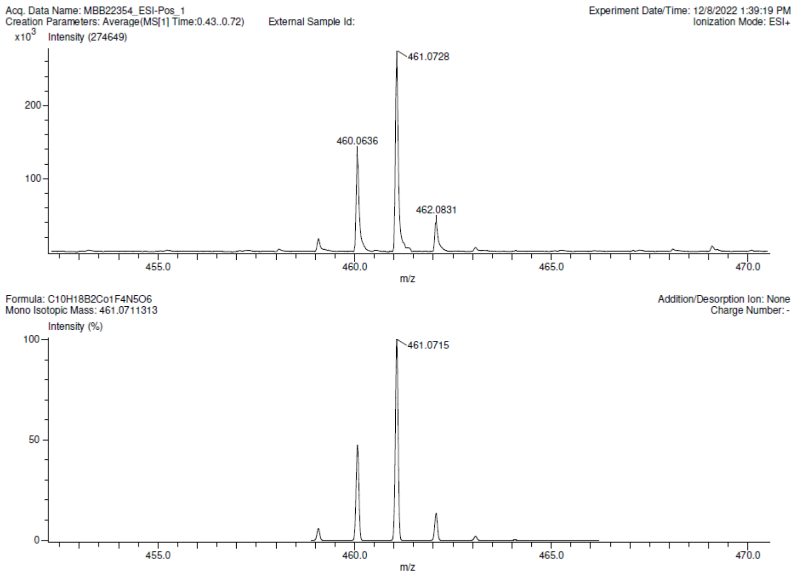

Figure SI17. HR-MS ESI spectrum of **6**, where at the top the measured spectrum is shown and at the bottom the simulated spectrum is given.

To investigate the samples for impurities that could influence the electrocatalytic response, the cyclic voltammogram of **6** (Figure SI18) was compared with earlier reported data.[21] We observed two peaks around the $Co^{II/I}$ redox couple, where only one would be expected. By spiking the sample with 20 μL H_2O (Milli-Q), it became clear that the second peak originates from exchange of the axial acetonitrile ligands by aquo ligands (light grey line). The further redox response of **6** was in excellent agreement with literature.[21]

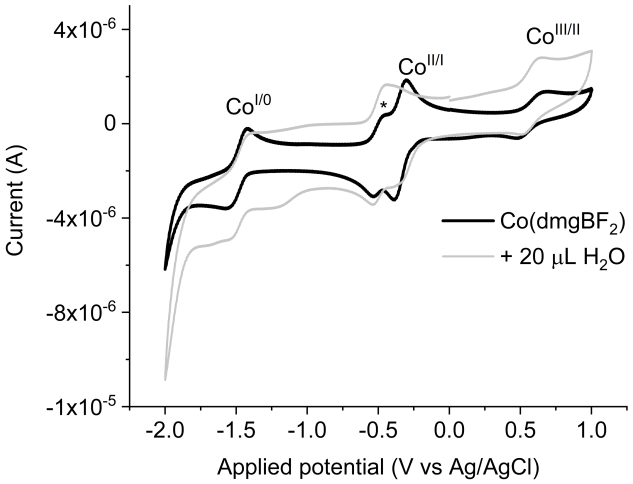

Figure SI18. Cyclic voltammogram (IUPAC plotting convention) of **6** (1 mM) in dry MeCN (0.1 M $TBABF_4$) purged with Ar. WE = glassy carbon, CE = Pt wire (d = 0.5 mm), RE = Ag/AgCl (in 3 M KCl) in an undivided cell, without IR drop compensation. The second scans are shown, starting at 0.0 V, scanning towards anodic potential with $v = 0.1\ V{\cdot}s^{-1}$. Part of the axial acetonitrile ligands exchanged for aquo ligands, indicated with an asterisk.

2. Other Co^{II} polypyridyl complexes and their mechanisms

To investigate common proton reduction mechanisms for cobalt polypyridyl complexes, we investigated numerous reported mechanistic studies, after which we compiled an overview of possible mechanistic pathways for Co^{II}-polypyridyl catalyzed H_2 evolution (Scheme SI2). These paths are based on the mechanistic studies described in Table SI5, which were an important inspiration for this work. In this table, we distinguish between electrochemical, photoelectrochemical (using a photosensitizer and sacrificial electron donors), chemical (using only sacrificial electron donors) and theoretical studies. We also describe the experimental conditions of the mechanistic study, and the mechanism (using the 'E', 'C' and 'EC' steps as explained in the main text).

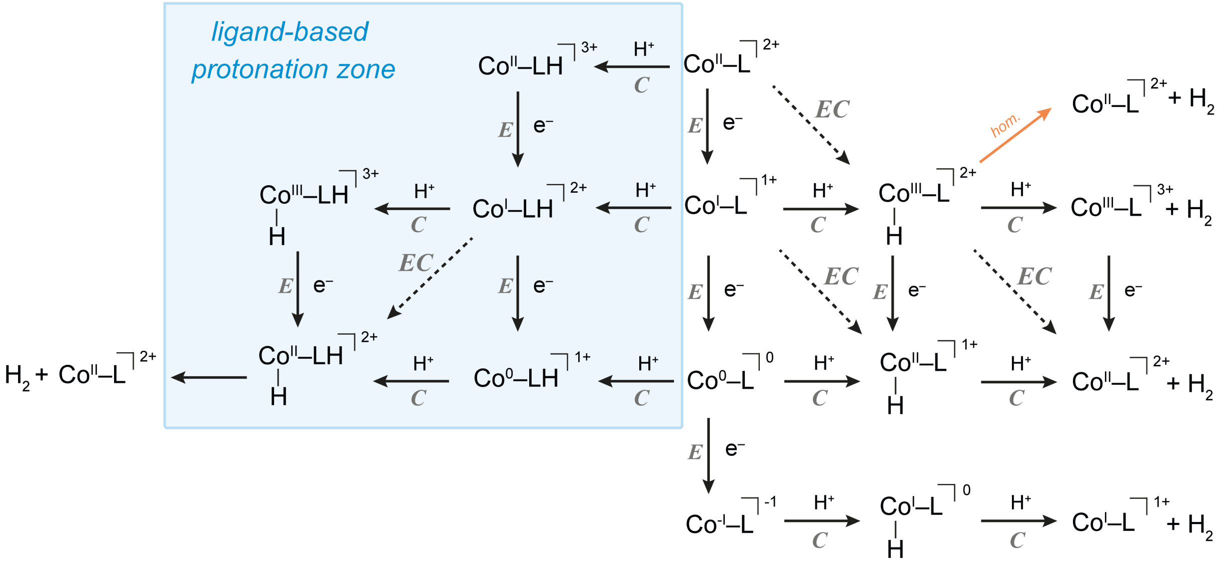

Scheme SI2. An overview of previously reported Co^{II}-polypyridyl mechanistic pathways based on numerous earlier mechanistic studies (see Table SI5), and the pathways mentioned in the main text of this article. On the right side, pathways are displayed that involve catalyst without ligands that can function as proton relay. On the left, in the blue panel, mechanisms that involve ligand protonation are indicated. Homolytic (hom) pathways are indicated with orange arrows, proton-coupled electron transfers (EC) are indicated with dashed arrows, electron transfer steps (E) are indicated with e$^-$, and protonation steps (C) with H$^+$.

Table SI5. Comparison of mechanisms between various cobalt-polypyridyl complexes.

Catalyst	Photochemical/ electrochemical/ chemical/ computational	Proton source	Mechanism	Reference
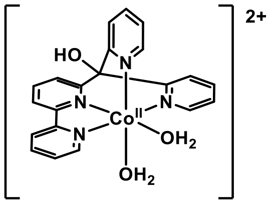 Co^{II}(aTPy) + derivatives	Photochemical	BRB buffer, pH >7	E(EC)C	22
	Electrochemical	BRB buffer, pH <7	(EC)EC	22
	Theoretical	water	ECEC	19
	Photochemical	TEOA in water, pH 8–8.5	ECEC	15

	Electrochemical	Water, pH 8	E(EC)C	23
	Electrochemical	Water, pH < 7	(EC)EC	23
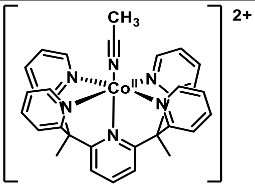 $Co^{II}(PY5Me_2)$	Electrochemical	Acetic acid in acetonitrile	After acetate binding: ECCE or EC(hom)	24
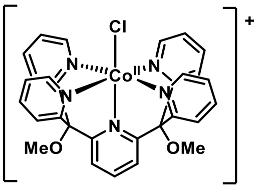 $Co^{II}(PY5)$	Electrochemical	Up to 5 eq. trifluoroacetic acid in acetonitrile	(EC)EC	25
	Electrochemical	From 6–16 eq. trifluoroacetic acid in acetonitrile	(EC)CE	25
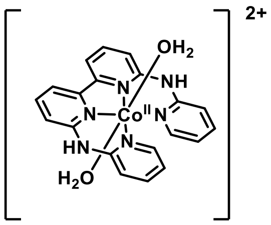 $Co^{II}(bapbpy)$	Chemical	Tris-HCl buffered aqueous solution (pH 7)	ECEC	26
	Electrochemical	HBF_4 in dimethylformamide	CECE	27
	Electrochemical	$HNEt_3BF_4$ in dimethylformamide	ECEC	27
	Electrochemical	Acetic acid in dimethylformamide	EECC	27
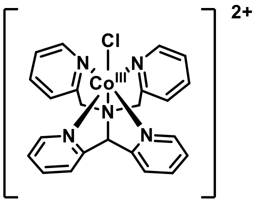 $Co^{III}(N_4Py)$	Photochemical	Ascorbic acid in 0.3 M NaCl aqueous solution	$EEC_{lig}C$ – first protonation takes place on pyridine	28
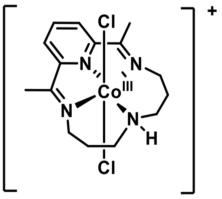 $Co^{III}(CR)$ + derivatives	Photochemical	Ascorbic acid in water	EC-hom or ECEC	29
	Electrochemical	0.1 M phosphate buffer, pH 2.2	PCET to Co^{II} with protonated amine → $C_{lig}EEC$	30
	Electrochemical	Aqueous phosphate (pH 7.0 and 2.0) or ascorbate (pH 4.1) buffer	After reduction to Co^{II}: ECCE	31
	Photochemical	Ascorbic acid in water (pH 4.1)	After reduction to Co^{II}: EECC	31

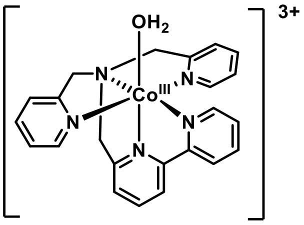 Co^{III}(DPA-bpy) + derivatives	Photochemical	Ascorbic acid in water (pH 4.0)	After reduction to Co^{II}: ECEC	32
	Theoretical	n.a.	After reduction to Co^{II}: ECEC	33
	Electrochemical/ photochemical	Aqueous phosphate buffer (varying pH)	E(C)CEC, where (C) is a structural reorganization within the catalyst	16
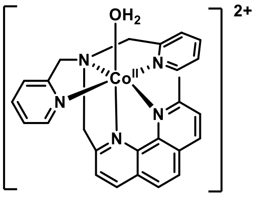 Co^{II}(DPA-Dmphen)	Photochemical/ electrochemical	Acetonitrile-water mixtures (pH 9–12)	ECEC	34
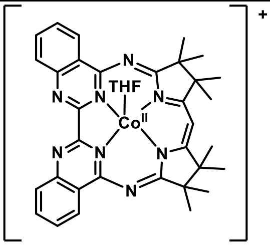 Co^{II}(Mabiq)	Electrochemical	*p*-cyanoanilinium in acetonitrile	ECEC	35
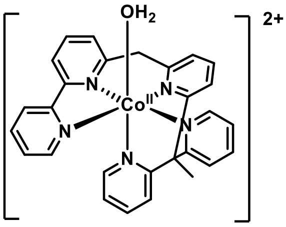 Co^{II}(Py3Me-BPy)	Theoretical	n.a.	ECEC	36
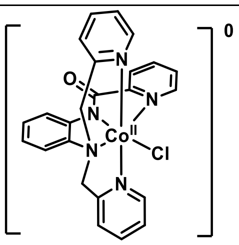 $Co^{II}(N_2N^{py}{}_3)$	Theoretical	n.a.	ECEC	37
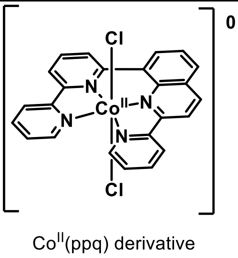 Co^{II}(ppq) derivative	Electrochemical/ theoretical	Aqueous phosphate buffer (pH 3.5–6.5)	E(EC)C	38

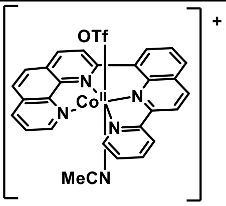 Co^{II}(ppq) derivative	Electrochemical	$HNEt_3BF_4$ in DMF	After reduction to Co^{I}: EECC	39
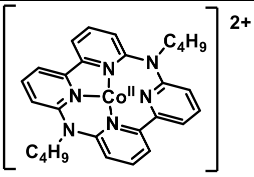 Co^{II}C-CAT	Electrochemical/ photochemical	*p*-cyanoanilinium in dimethyl-formamide (electrochemical) and aqueous ascorbic acid buffer (pH 4.5)	$C_{lig}EE_{lig}C$	40
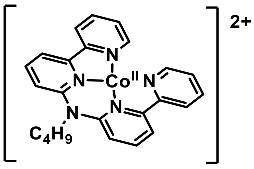 Co^{II}O-CAT				
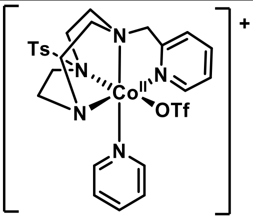 Co^{II}(Py$_2{}^{Ts}$tacn)	Electrochemical	Trifluoroacetic acid in acetonitrile or water	ECCE	41
	Photochemical	NEt_3 in water-acetonitrile mixture (7:3)	ECEC	41
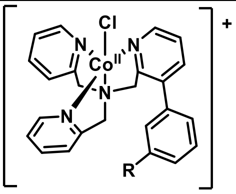 Co^{II}(TMPA)	Electrochemical	Trifluoroacetic acid in acetonitrile	(EC)EC	42
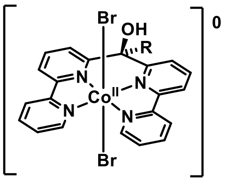 Co^{II}(bpy$_2$CO) derivative R = Ph, Me	Electrochemical	$HNEt_3BF_4$ in dimethylformamide	After reduction to Co^{I}: EECC	43
	Electrochemical	Aqueous BRB solution, pH > 6	After reduction to Co^{I}: EECC	43
	Electrochemical	Aqueous BRB solution, pH 2–6	EECC	43

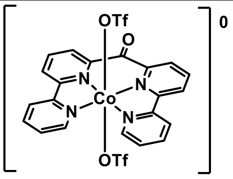 Co(bpy_2CO) 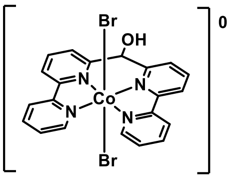 Co(bpy_2COH) Co(bpy_2CO) converts to Co(bpy_2COH) under reductive conditions	Electrochemical	$HNEt_3BF_4$ in dimethylformamide	After reduction to Co^I: EECC	43
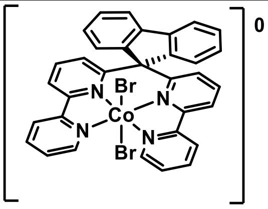	Electrochemical	Aqueous BRB solution pH < 7	E(EC)C	43
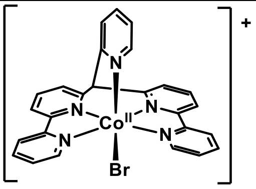 Co^{II}(aPPy)	Electrochemical	Aqueous BRB solution, pH 2–9	EECC	43

3. Acetic acid as proton source

Acetic acid was not included in the mechanistic study, since its influence on the redox properties of the catalyst seemed to extend beyond being an innocent proton source, based on the shift of the $Co^{II/I}$ redox potential (see Figure SI19). Such an influence of acetic acid was observed before for the $Co(PY5Me_2)$ complex reported by Long and co-workers.[24] In their case, complexation of acetate to a pentapyridyl Co^{II} complex also generated a new species, with a 270 mV more negative reduction potential than the $Co^{II/I}$ couple. A similar cathodic shift of ~230 mV is observed for the [Co(aPPy)Br]Br complex. Due to the unfavorable higher overpotential for proton reduction, no further mechanistic studies were carried out.

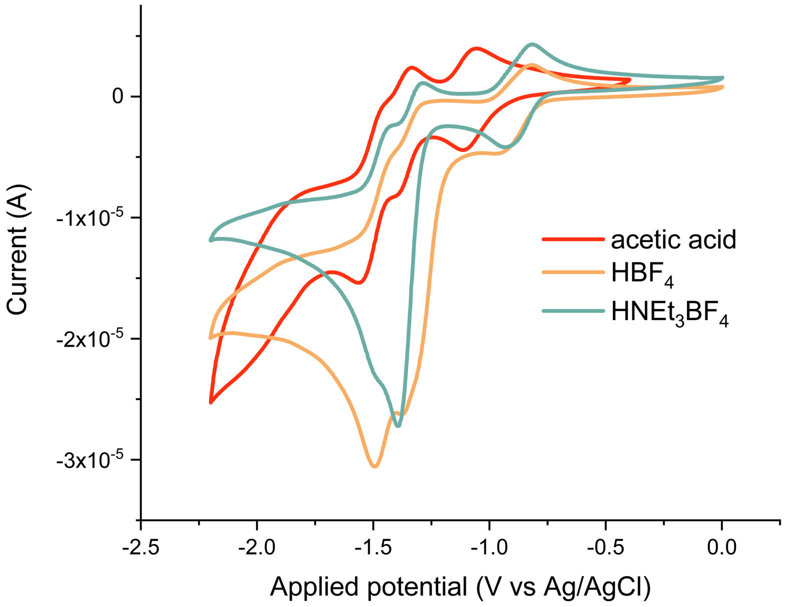

Figure SI19. Cyclic voltammograms (IUPAC plotting convention) of [Co(aPPy)Br]Br (1 mM) with different acid sources (1 mM) in dry DMF (0.1 M $TBABF_4$) purged with Ar. WE = glassy carbon, CE = Pt wire (d = 0.5 mm), RE = Ag/AgCl (in 3 M KCl) in an undivided cell, without IR drop compensation. The second scans are shown, starting at –0.1 V or –0.4 V, scanning towards cathodic potential with $v = 0.1\ V\cdot s^{-1}$.

4. Additional notes on HBF_4 as proton source

The CVs in presence of more than one equivalent HBF_4 showed a loss of redox features from the start of the CV measurements (see Figure SI20). This indicates that the Co(aPPy) complex itself is not stable towards more than one equivalent of HBF_4, even before reduction.

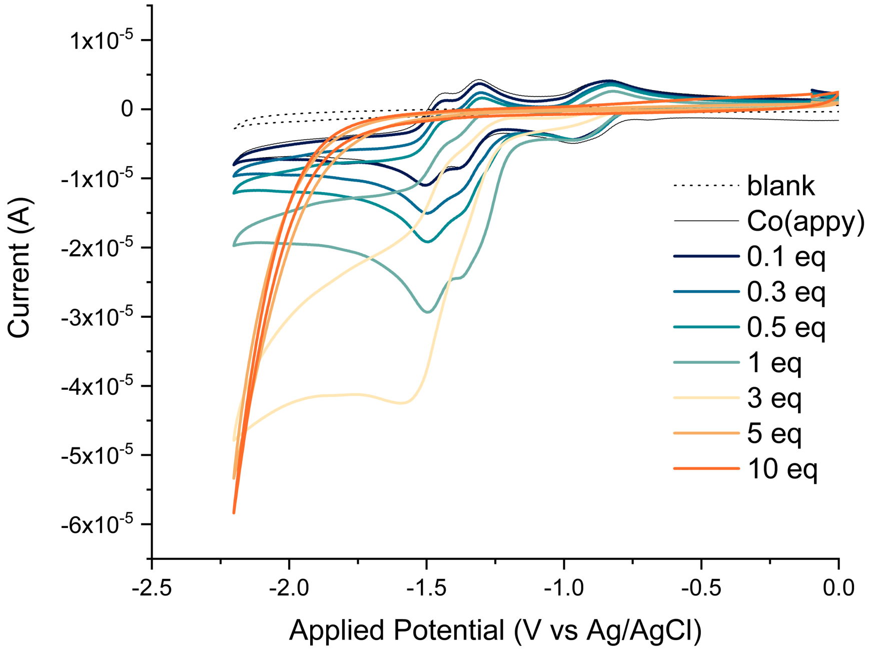

Figure SI20. Cyclic voltammograms (IUPAC plotting convention) of [Co(aPPy)Br]Br (1 mM) with increasing HBF_4 concentration in dry DMF (0.1 M $TBABF_4$) purged with Ar, WE = glassy carbon, CE = Pt wire (d = 0.5 mm), RE = Ag/AgCl (in 3 M KCl) in an undivided cell, without IR drop compensation. The first scans are shown, starting at –0.1 V, scanning towards cathodic potential with v = 0.1 $V \cdot s^{-1}$. The equivalents of HBF_4 are given with respect to the catalyst.

5. Screening of buffer solutions

For pH-dependent cyclic voltammetry studies in an aqueous system, the Britton-Robinson buffer (BRB) is commonly used.[23,43] However, this buffer consist of three different acids, namely acetic, phosphoric and boric acid. As became apparent from the various shoulders in the catalytic regime, as well as multiple oxidative peaks in the backward scans, the catalyst might respond differently to all three acids, which complicates the interpretation of the voltammograms (Figure SI21). The nature of these different interactions was not further investigated, but it has been reported before that for instance acetic acid can ligate to Co^{II} polypyridyl complexes.[24] It should be noted that the flat line at pH 4.5 (in both measurements of the duplo) is caused by a generated hydrogen bubble covering the electrode and preventing contact with the electrolyte, leading to loss of signal.

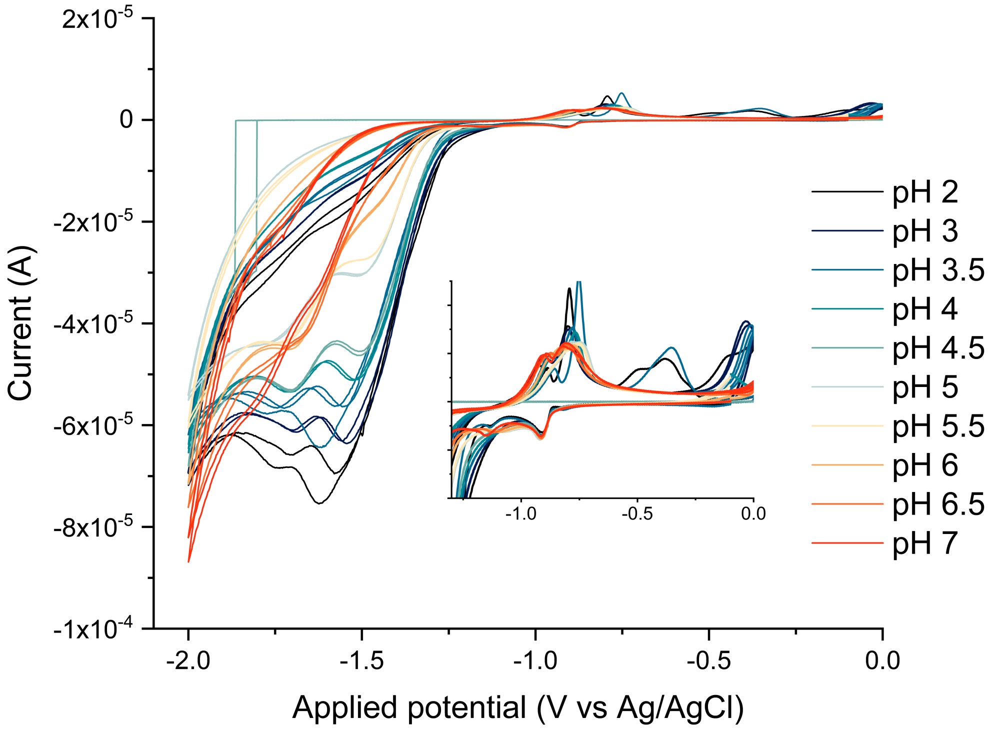

Figure SI21. Cyclic voltammogram (IUPAC plotting convention), in duplo, of 1 mM [Co(aPPy)Br]Br BRB buffer at varying pH (40 mM phosphoric acid, 40 mM boric acid and 40 mM acetic acid in milli-Q, stepwise basified with 5 M NaOH (99.99%), and 0.1 M $NaBF_4$ as electrolyte). WE = HMDE, CE = carbon rod and RE = Ag/AgCl (in 3 M KCl) were used in an undivided cell, without IR drop compensation. The second scans are shown, which started at –0.1 V, after which the potential swept in the cathodic direction with $v = 0.5\ V \cdot s^{-1}$.

Therefore, various other buffer solutions were investigated, including phosphate (Figure SI22), ascorbate (Figure SI23) and citric (main text). The latter was chosen as the preferred system, since this acid can efficiently buffer the pH range of interest (pH 3–6.2)[44] and results in a less complex redox response compared to the BRB solution.

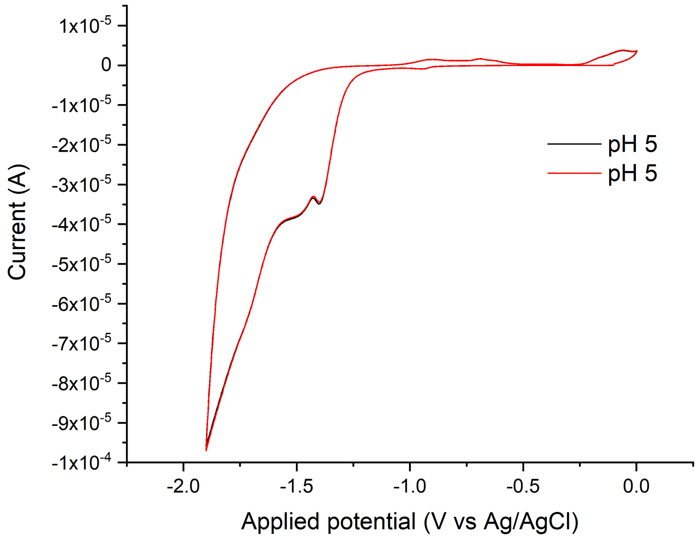

Figure SI22. Cyclic voltammogram (IUPAC plotting convention), in duplo, of 1 mM [Co(aPPy)Br]Br in pH 5 ascorbate buffer (0.2 M sodium ascorbate, basified with 5 M NaOH (99.99%), and 0.1 M $NaBF_4$ as electrolyte, in milli-Q). WE = HMDE, CE = carbon rod and RE = Ag/AgCl (in 3 M KCl) were used in an undivided cell, without IR drop compensation. The second scans are shown, which started at –0.1 V, after which the potential swept in the cathodic direction with $v = 0.5\ V{\cdot}s^{-1}$.

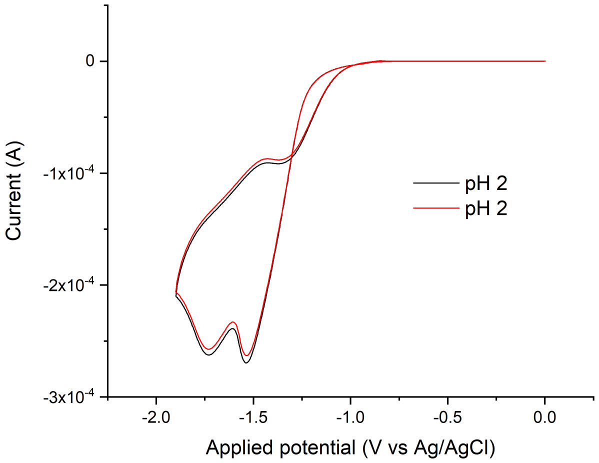

Figure SI23. Cyclic voltammogram (IUPAC plotting convention), in duplo, of 1 mM [Co(aPPy)Br]Br in pH 2 phosphate buffer (0.2 M sodium phosphate, basified with 5 M NaOH (99.99%), and 0.1 M $NaBF_4$ as electrolyte, in milli-Q). WE = HMDE, CE = carbon rod and RE = Ag/AgCl (in 3 M KCl) were used in an undivided cell, without IR drop compensation. The second scans are shown, which started at –0.1 V, after which the potential swept in the cathodic direction with $v = 0.5\ V{\cdot}s^{-1}$.

6. [Co(aPPy)Br]Br compared to [Co(aPPy)(H_2O)](ClO_4)$_2$

The redox response of [Co(aPPy)Br]Br and [Co(aPPy)](ClO_4)$_2$ in presence of acid are similar (Figure SI24). Since the latter complex contains a non-coordinating anion, and their response is similar, it is assumed that the bromide ion dissociates in solution.

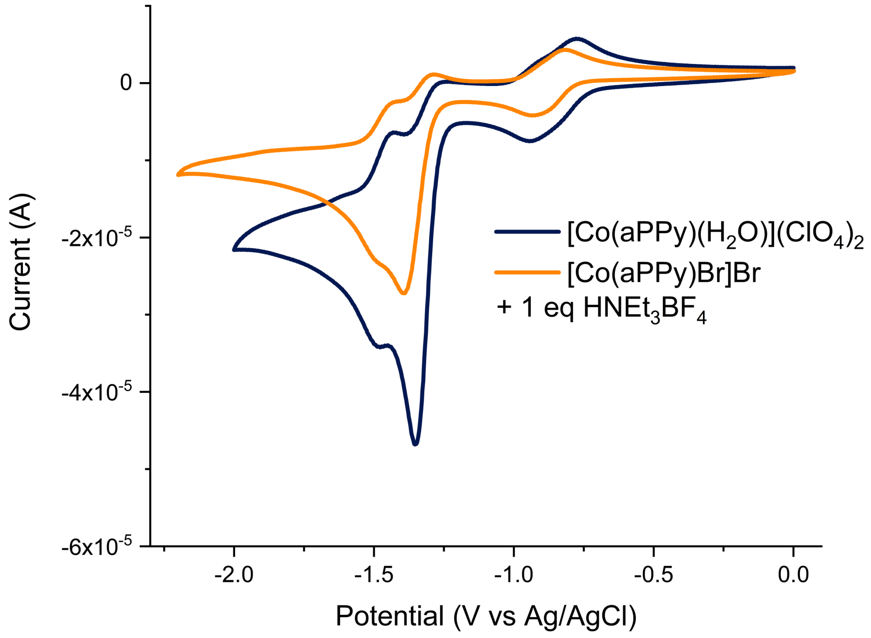

Figure SI24. Cyclic voltammograms (IUPAC plotting convention) of [Co(aPPy)Br]Br (1 mM in DMF, containing 0.1 M $TBABF_4$) and [Co(aPPy)](ClO_4)$_2$ (1 mM in MeCN, containing 0.1 M $TBABF_4$), in presence of 1 mM $HNEt_3BF_4$. The electrolyte solutions were purged with Ar. WE = glassy carbon, CE = Pt wire, RE = Ag/AgCl (in 3 M KCl) in an undivided cell, without IR drop compensation. The second scans are shown, starting at –0.1 V, scanning towards cathodic potentials with v = 0.1 V·s^{-1}.

7. The transformation of the Co(aPPy) precatalyst to a different species

A first indication was a change in dependency of the potential at a certain catalytic current with respect to the pH. This potential starts to show a more negative linear relation over multiple scans (Figure 5, main text). Furthermore, upon scanning backwards in the first scan, line crossing was observed, as is indicated with the red circles in Figure SI25.

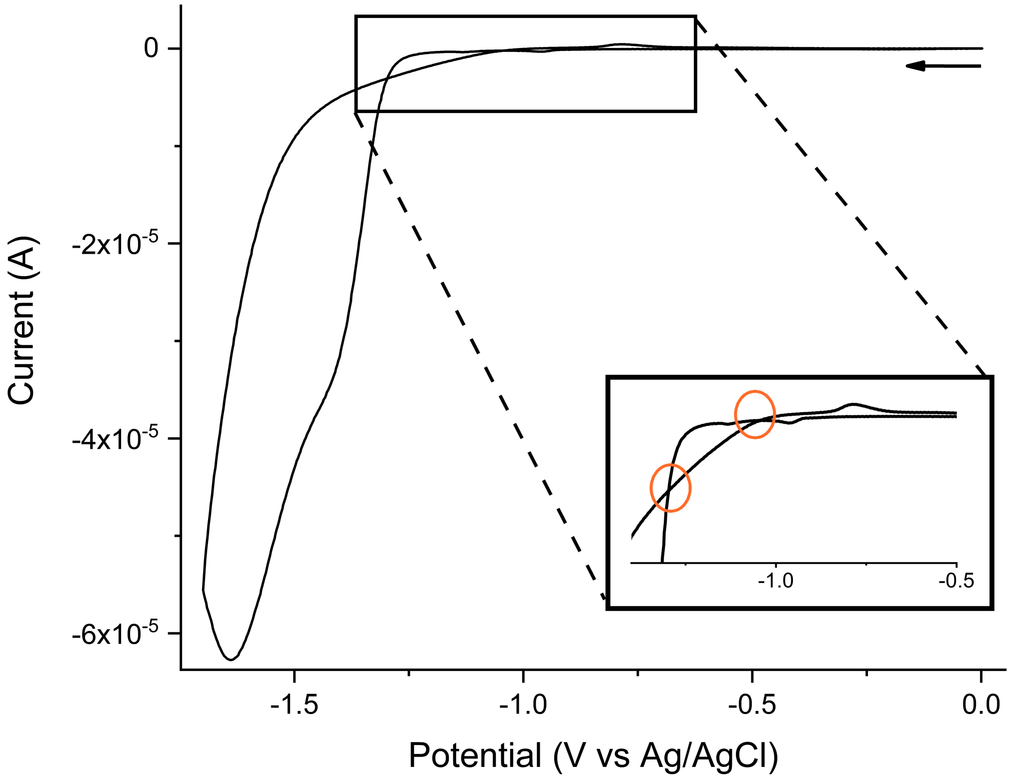

Figure SI25. Cyclic voltammogram (IUPAC plotting convention) of 1 mM [Co(aPPy)Br]Br in pH 4.5 citric acid buffer (0.2 M sodium citrate, basified with 5 M NaOH (99.99%), and 0.1 M $NaBF_4$ as electrolyte in Milli-Q). WE = HMDE, CE = carbon rod and RE = Ag/AgCl (in 3 M KCl) were used in an undivided cell, without IR drop compensation. The first scan is shown, which initiated at –0.1 V, after which the potential swept in the cathodic direction with v = 0.1 V·s^{-1} (as indicated by the black arrow).

8. Ruling out adsorption of the complex onto the Hg electrode

Randles-Sevcik analysis of the $Co^{II/I}$ reduction peak in the second scan was carried out to investigate whether the catalytic species was homogeneous or heterogenized on the surface. The square root of the scan rate was plotted against the peak current of the reductive redox event (*i2*). A linear fit to the points indicate a homogeneously diffusing species. Further peaks were not investigated since their presence and position greatly depended on the scan rate, which complicates comparison. At all pH values, a linear function could be fitted to the points between $v = 0.1$ to 1 $V{\cdot}s^{-1}$, with an R-squared value of 0.99 or higher (see Figure SI26).

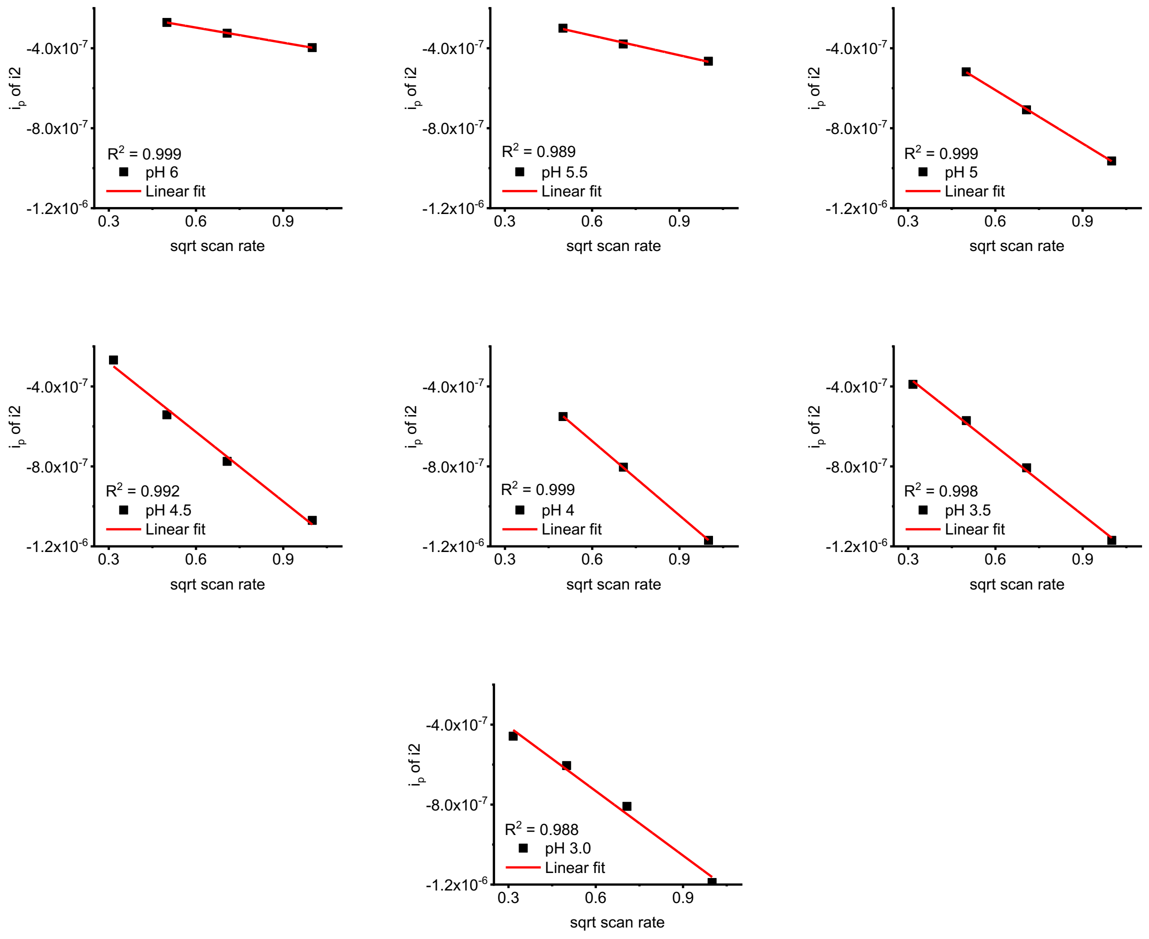

Figure SI26. Randles-Sevcik analyses of the $Co^{II/I}$ reduction peak (in the second CV cycle) at point *i2* (main text) from pH 3–6.

9. Mononuclear or binuclear catalysis

Proton reduction can proceed via a mononuclear or binuclear path, where in the latter case two metal hydride intermediates can combine to generate hydrogen. To investigate via which pathway catalysis likely proceeds, the electrochemical behavior of the catalyst while varying its concentration can be studied, to determine the reaction order in catalyst. Bulk electrolysis was performed in a 10 mL solution of 0.5 mM Co(aPPy) in Milli-Q, containing 0.1 M $NaBF_4$ as supporting electrolyte and 0.2 M citric acid (the solution pH was increased to pH 4 using 5 M NaOH) in an undivided cell. The bulk electrolyses at lower concentrations of Co(aPPy) were carried out by further diluting this solution with a pH 4 stock solution of 0.1 M $NaBF_4$ and 0.2 M citric acid. A potential of –1.9 V versus Ag/AgCl was applied for 300 seconds, while monitoring the current and accumulated charge over time. The total charge after 300 s was plotted against the catalyst concentration, as shown in Figure SI27. In addition, the *Q*–*t* plots are displayed in Figure SI28.

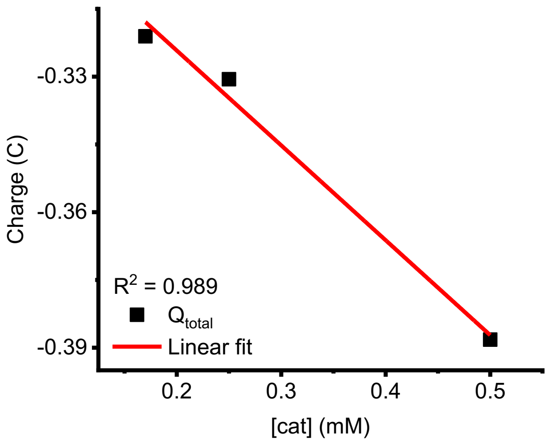

Figure SI27. The total charge that passed through the electrode during constant potential bulk electrolysis in an undivided cell vs varying Co(aPPy) concentrations in 0.1 M $NaBF_4$ and 0.2 M citric acid buffer in Milli-Q (pH 4). A dropping mercury electrode was used as WE, in combination with a carbon rod CE and a Ag/AgCl (in 3 M KCl) RE, and a potential of –1.9 V was applied for 300 s while stirring the reaction at 3000 rpm.

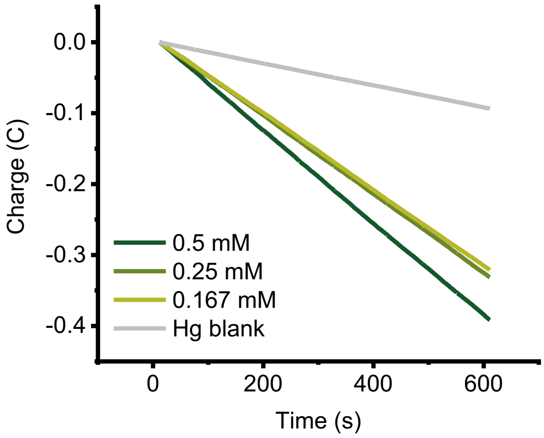

Figure SI28. Charge vs time plots of the constant potential bulk electrolysis in an undivided cell at varying Co(aPPy) concentrations in 0.1 M $NaBF_4$ and 0.2 M citric acid buffer in milli-Q (pH 4). A dropping mercury electrode was used as WE, in combination with a carbon rod CE and a Ag/AgCl (in 3 M KCl) RE, and a potential of –1.9 V was applied while stirring the reaction at 3000 rpm.

10. Constant potential electrolysis with Co(aPPy) and Co(dmgBF$_2$)$_2$

To determine the electrochemical stability and activity over longer periods of time, bulk electrolysis was carried out using a custom-made H–cell. To compare the proton reduction activity of the bare Hg electrode, Co(aPPy) and Co(dmgBF$_2$)$_2$, we carried out constant potential electrolysis at –1.2 V versus Ag/AgCl for 3600 s, during which we monitored the current and charge over time (see Figure SI29 for the *i* vs *t* plots).

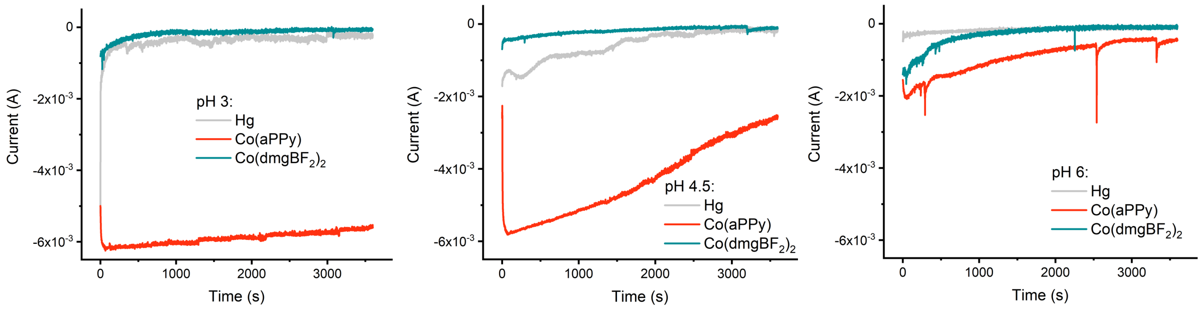

Figure SI29. Current vs time plots of the bulk electrolyses at pH 3 (left), pH 4.5 (middle) and pH 6 (right) in 0.1 M Na_2SO_4 and 0.1 M citric acid buffer in Milli-Q.

The low stability of the Co(dmgBF$_2$)$_2$ complex was indicated, firstly, by the observed current being lower in presence of catalyst than in its absence (Figure SI29). The decomposed catalyst thus suppresses the activity of the bare mercury electrode (except for the first 1000 s at pH 6). Secondly, a black layer (amalgam) formed on the surface of the Hg pool electrode in presence of Co(dmgBF$_2$)$_2$, whereas this was not the case in the blank experiments or the experiments in presence of Co(aPPy), as shown in Figure SI30.

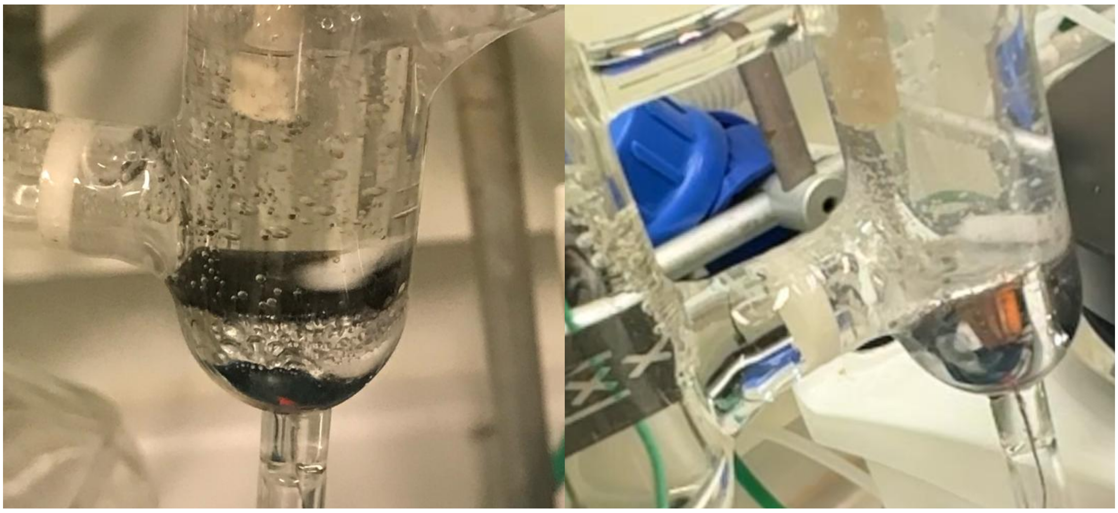

Figure SI30. Images of the mercury pool working electrode during electrolysis bulk electrolysis, with amalgam formation shown on the left (in presence of Co(dmgBF$_2$)$_2$) and the bare Hg surface shown on the right.

We assessed the catalyst stability of Co(aPPy) by comparing the observed currents at 30 and 60 minutes (Figure SI29 and Table SI6). We chose these points, as earlier points only represent the catalytic activity to a limited extent. For instance, at the beginning of the electrolysis, high currents can be related to a high initial proton concentration at the electrode surface, which drops during catalysis, as the protons are converted to H_2 and need to be replenished. Furthermore, as hydrogen bubbles are generated, they can stick to the Hg

pool, but in the beginning, these H_2 bubbles are not there yet, which could also result in higher initial currents. For those reasons, we chose thus the two points at 30 and 60 minutes, where we assume that the system has equilibrated by then. Furthermore, the catalytic TON after 3600 s was derived from the charge that passed through the cell, using the values in Table SI6.

Table SI6. Values derived from the CPE at –1.2 V vs Ag/AgCl that were used to calculate the decrease in catalytic current (see row "*decrease in* $i_{Co(aPPy)}$") and the TON (bottom row) at different pH values.

	pH 3	pH 4.5	pH 6
i_{Hg} 1800 s (A)	2.018E–4	–4.44214E–4	–1.15509E–4
i_{Hg} 3600 s(A)	2.293E–4	–1.76697E–4	–1.17249E–4
Q_{Hg} 3600 s (C)	–1.32084	–2.03089	–0.58934
$i_{Co(aPPy)}$ 1800 s (A)	0.00589	–0.00448	–8.05359E–4
corrected $i_{Co(aPPy)}$ 1800 s (A)	0.00569	–0.00404	–6.8985E–4
$i_{Co(aPPy)}$ 3600 s (A)	0.00561	–0.00256	–4.47113E–4
corrected $i_{Co(aPPy)}$ 3600 s (A)	0.00538	–0.00238	–3.29865E–4
decrease in $i_{Co(aPPy)}$ (%)	5	41	52
$Q_{Co(aPPy)}$ 3600 s (C)	–21.2879	–15.51849	–3.33014
corrected $Q_{Co(aPPy)}$ 3600 s (C)	–19.96706	–13.4876	–2.7408
$TON_{Co(aPPy)}$ 3600 s	2586.80883	1747.37035	355.08073

Long term constant potential electrolysis at –1.2 V versus Ag/AgCl was carried out to compare the electrocatalytic TON_{max} of Co(aPPy) to the reported value under photocatalytic conditions. The amperogram is given in Figure SI31, and the relevant values and calculations are provided in Table SI7.

Finally, we investigated the influence of the Co^I intermediate on the catalyst stability with chrono amperometry at varying potential. The potential was kept at –1.20 V for 20 minutes, changed to –1.00 V (–0.95 V for pH 3 due to the earlier catalytic onset) for 20 min, followed by another 20 minutes at –1.20 V. The corresponding amperograms are given in Figure 8. We have compared the decrease in current between points A and B for both runs with and without the interval at –1.0 V, for which the values are given in Table SI8. At pH 4.5, the decrease in activity was 49% for the CPE at –1.2 V, whereas the current only reduced by 32% with the –1.0 V interval. At pH 6.0, the activity decreased 58% and 55%, with and without the interval, respectively.

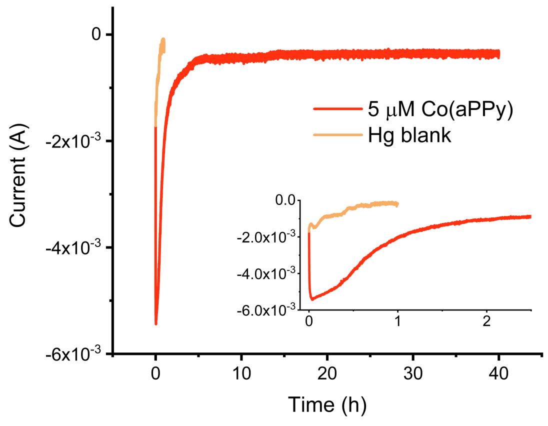

Figure SI31. Current vs time plot of the 40 h bulk electrolysis at –1.2 V vs Ag/AgCl 5 µM Co(aPPy) at pH 4.5 in 0.1 M Na_2SO_4 and 0.1 M citric acid buffer in Milli-Q.

Table SI7. Values derived from the constant potential electrolysis at –1.2 V vs Ag/AgCl that were used to calculate the electrocatalytic TON_{max} at pH 4.5.

	Hg	Co(aPPy)	Co(aPPy) corrected
i 3600 s	-1.76697E-4		
Q 3600 s	-2.03089		
Measured Q_{total} 144 000 s		-74.01006	
Extrapolated Q_{total} 144 000 s	-26.83912[a]		-47.17094
$TON_{Co(aPPy)}$ 144 000 s			**6111**[b]

a) we extrapolated the current measured at 3600 s to calculate this value, via the formula: $Q_{total} = Q_{3600} + (i_{3600} \cdot 140400)$. b) calculated via the formula: $TON = Q_{total} / (F \cdot z \cdot n_{cat})$, where F = the faraday constant, z is the number of electrons of the half-reaction (z = 2), n_{cat} is moles of catalyst present ($4 \cdot 10^{-8}$ mol).

Table SI8. The currents at point A and B (in Figure 8) and the calculated decrease in current for the chrono amperometry experiments at pH 4.5 and 6.0.

	Current point A (mA)	Current point B (mA)	Decrease in current (%)
pH 4.5, no interval	-4.997	-2.555	49
pH 4.5, with interval	-5.014	-3.408	32
pH 6.0, no interval	-1.009	-0.447	55
pH 6.0, with interval	-1.680	-0.700	58

11. UV-vis titration of the Co(aPPy) complex from acidic to basic pH

[Co(aPPy)Br]Br (1.9 mg) was dissolved in 2 mL 0.1 M Na_2SO_4 + 0.1 M citric acid in Milli-Q (pH was adjusted to 3 before adding the catalyst), resulting in a 2 mM solution. The pH of the solution was increased stepwise with a 5 M NaOH solution (in Milli-Q), and the solution was vigorously shaken before the UV-vis response was measured (Figure SI32). A blank pH 3 buffer solution (0.1 M Na_2SO_4 and 0.1 M citric acid in Milli-Q) was used as blank, and we confirmed that the visible light absorption of this solution remained unchanged over the full pH scale, as blank before (pH 3) versus blank after (pH 12.1) are identical. Nevertheless, as the pH increased in presence of Co(aPPy), we see a clear increase in the baseline of the absorption spectrum, with concomitant formation of a precipitate in the cuvette. Upon decreasing the pH to 3 again, the baseline does not restore to its original position, even not after four days. We also observed the rise of a reversible absorption below 450 nm upon increasing the pH from 5.2 to 6.3, which is potentially correlated to the reversible protonation of the pyridine ligand.

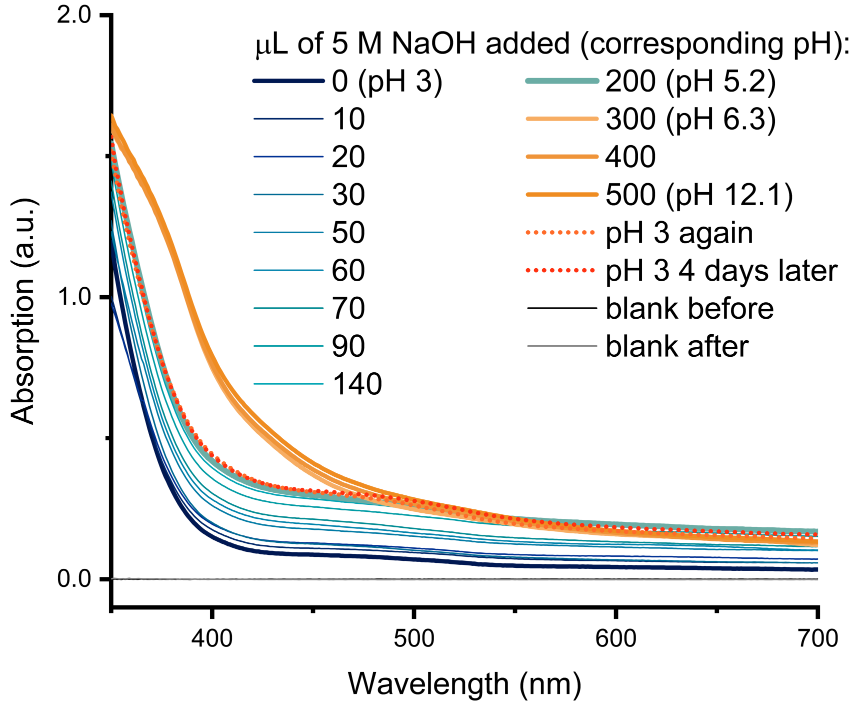

Figure SI32. UV-vis titration of the Co(aPPy) complex (2 µM) in aqueous citric acid buffer (0.1 M citric acid, 0.1 M Na_2SO_4).

References

1. Wang, C.; Guo, L.; Li, H.; Wang, Y.; Weng, J.; Wu, L. Preparation of Simple Ammonium Ionic Liquids and Their Application in the Cracking of Dialkoxypropanes. *Green Chem.* **2006**, 8 (7), 603–607. https://doi.org/10.1039/b600041j.
2. Fulmer, G. R.; Miller, A. J. M.; Sherden, N. H.; Gottlieb, H. E.; Nudelman, A.; Stoltz, B. M.; Bercaw, J. E.; Goldberg, K. I. NMR Chemical Shifts of Trace Impurities: Common Laboratory Solvents, Organics, and Gases in Deuterated Solvents Relevant to the Organometallic Chemist. *Organometallics* **2010**, 29 (9), 2176–2179. https://doi.org/10.1021/om100106e.
3. Stoll, S.; Schweiger, A. EasySpin, a Comprehensive Software Package for Spectral Simulation and Analysis in EPR. *J. Magn. Reson.* **2006**, 178 (1), 42–55. https://doi.org/10.1016/j.jmr.2005.08.013.
4. Casey, T. cwEPR MATLAB Central File Exchange https://www.mathworks.com/matlabcentral/fileexchange/73292-cwepr (accessed Jul 8, 2022).
5. Bruker. APEX2 Software. Madison WI, USA 2014.
6. Sheldrick, G. M. SADABS. University of Göttingen, Germany 2008.

7. Sheldrick, G. M. Crystal Structure Refinement with SHELXL. *Acta Crystallogr.* **2015**, 71, 3–8.
8. Sheldrick, G. M. SHELXL2013. University of Göttingen, Germany 2013.
9. Krejčik, M.; Daněk, M.; Hartl, F. Simple Construction of an Infrared Optically Transparent Thin-Layer Electrochemical Cell. *J. Electroanal. Chem. Interfacial Electrochem.* **1991**, 317 (1–2), 179–187. https://doi.org/10.1016/0022-0728(91)85012-e.
10. Bachmann, C.; Guttentag, M.; Spingler, B.; Alberto, R. 3d Element Complexes of Pentadentate Bipyridine-Pyridine-Based Ligand Scaffolds: Structures and Photocatalytic Activities. *Inorg. Chem.* **2013**, 52, 6055. https://doi.org/10.1021/ic4004017.
11. Hoang, T. N. Y.; Humbert-Droz, M.; Dutronc, T.; Guénée, L.; Besnard, C.; Piguet, C. A Polyaromatic Terdentate Binding Unit with Fused 5,6-Membered Chelates for Complexing s-, p-, d-, and f-Block Cations. *Inorg. Chem.* **2013**, 52 (9), 5570–5580. https://doi.org/10.1021/ic400526j.
12. Wolpher, H.; Johansson, O.; Abrahamsson, M.; Kritikos, M.; Sun, L.; Åkermark, B. A Tridentate Ligand for Preparation of Bisterpyridine-like Ruthenium(II) Complexes with an Increased Excited State Lifetime. *Inorg. Chem. Commun.* **2004**, 7 (3), 337–340. https://doi.org/10.1016/j.inoche.2003.12.007.
13. England, J.; Bill, E.; Weyhermüller, T.; Neese, F.; Atanasov, M.; Wieghardt, K. Molecular and Electronic Structures of Homoleptic Six-Coordinate Cobalt(I) Complexes of 2,2′:6′,2″-Terpyridine, 2,2′-Bipyridine, and 1,10-Phenanthroline. An Experimental and Computational Study. *Inorg. Chem.* **2015**, 54 (24), 12002–12018. https://doi.org/10.1021/acs.inorgchem.5b02415.
14. Schweinfurth, D.; Krzystek, J.; Atanasov, M.; Klein, J.; Hohloch, S.; Telser, J.; Demeshko, S.; Meyer, F.; Neese, F.; Sarkar, B. Tuning Magnetic Anisotropy Through Ligand Substitution in Five-Coordinate Co(II) Complexes. *Inorg. Chem.* **2017**, 56 (9), 5253–5265. https://doi.org/10.1021/acs.inorgchem.7b00371.
15. Rodenberg, A.; Orazietti, M.; Probst, B.; Bachmann, C.; Alberto, R.; Baldridge, K. K.; Hamm, P. Mechanism of Photocatalytic Hydrogen Generation by a Polypyridyl-Based Cobalt Catalyst in Aqueous Solution. *Inorg. Chem.* **2015**, 54 (2), 646–657. https://doi.org/10.1021/ic502591a.
16. Lewandowska-Andralojc, A.; Baine, T.; Zhao, X.; Muckerman, J. T.; Fujita, E.; Polyansky, D. E. Mechanistic Studies of Hydrogen Evolution in Aqueous Solution Catalyzed by a Tertpyridine–Amine Cobalt Complex. *Inorg. Chem.* **2015**, 54 (9), 4310–4321. https://doi.org/10.1021/ic5031137.
17. Singh, W. M.; Mirmohades, M.; Jane, R. T.; White, T. A.; Hammarström, L.; Thapper, A.; Lomoth, R.; Ott, S. Voltammetric and Spectroscopic Characterization of Early Intermediates in the Co(II)–Polypyridyl-Catalyzed Reduction of Water. *Chem. Commun.* **2013**, 49 (77), 8638–8640. https://doi.org/10.1039/c3cc44655g.
18. Lucarini, F.; Fize, J.; Morozan, A.; Marazzi, M.; Natali, M.; Pastore, M.; Artero, V.; Ruggi, A. Insights into the Mechanism of Photosynthetic H_2 Evolution Catalyzed by a Heptacoordinate Cobalt Complex. *Sustain. Energy Fuels* **2020**, 4 (2), 589–599. https://doi.org/10.1039/C9SE00434C.
19. Gurdal, Y.; Iannuzzi, M. Comparison of Penta and Tetra-Pyridyl Cobalt-Based Catalysts for Water Reduction: H_2 Production Cycle, Solvent Response and Reduction Free Energy. *ChemPhysChem* **2020**, 21 (24), 2692–2700. https://doi.org/10.1002/cphc.202000600.
20. Bakac, A.; Espenson, J. H. Unimolecular and Bimolecular Homolytic Reactions of Organochromium and Organocobalt Complexes. Kinetics and Equilibria. *J. Am. Chem. Soc.* **1984**, 106 (18), 5197–5202. https://doi.org/10.1021/ja00330a027.
21. Baffert, C.; Artero, V.; Fontecave, M. Cobaloximes as Functional Models for Hydrogenases. 2. Proton Electroreduction Catalyzed by Difluoroborylbis(Dimethylglyoximato)Cobalt(II) Complexes in Organic Media. *Inorg. Chem.* **2007**, 46 (5), 1817–1824. https://doi.org/10.1021/ic061625m.
22. Alberto, R.; Iannuzzi, M.; Gurdal, Y.; Probst, B. $[Co^{II}(BPyPy_2COH)(OH_2)_2]^{2+}$: A Catalytic Pourbaix Diagram and AIMD Simulations on Four Key Intermediates. *Chimia* **2019**, 73 (11), 906. https://doi.org/10.2533/chimia.2019.906.
23. Schnidrig, S.; Bachmann, C.; Müller, P.; Weder, N.; Spingler, B.; Joliat-Wick, E.; Mosberger, M.; Windisch, J.; Alberto, R.; Probst, B. Structure-Activity and Stability Relationships for Cobalt Polypyridyl-Based Hydrogen-Evolving Catalysts in Water. *ChemSusChem* **2017**, 10 (22), 4570–4580. https://doi.org/10.1002/cssc.201701511.
24. King, A. E.; Surendranath, Y.; Piro, N. A.; Bigi, J. P.; Long, J. R.; Chang, C. J. A Mechanistic Study of Proton Reduction Catalyzed by a Pentapyridine Cobalt Complex: Evidence for Involvement of an Anation-Based Pathway. *Chem. Sci.* **2013**, 4 (4), 1578–1587. https://doi.org/10.1039/c3sc22239j.
25. Deponti, E.; Luisa, A.; Natali, M.; Iengo, E.; Scandola, F. Photoinduced Hydrogen Evolution by a Pentapyridine Cobalt Complex: Elucidating Some Mechanistic Aspects. *Dalton Trans.* **2014**, 43 (43), 16345–16353. https://doi.org/10.1039/c4dt02269f.
26. Queyriaux, N.; Giannoudis, E.; Windle, C. D.; Roy, S.; Pécaut, J.; Coutsolelos, A. G.; Artero, V.; Chavarot-Kerlidou, M. A Noble Metal-Free Photocatalytic System Based on a Novel Cobalt Tetrapyridyl Catalyst for Hydrogen Production in Fully Aqueous Medium. *Sustain. Energy Fuels* **2018**, 2 (3), 553–557. https://doi.org/10.1039/C7SE00428A.
27. Queyriaux, N.; Sun, D.; Fize, J.; Pécaut, J.; Field, M. J.; Chavarot-Kerlidou, M.; Artero, V. Electrocatalytic Hydrogen Evolution with a Cobalt Complex Bearing Pendant Proton Relays: Acid Strength and Applied Potential Govern Mechanism and Stability. *J. Am. Chem. Soc.* **2020**, 142 (1), 274–282. https://doi.org/10.1021/jacs.9b10407.

28. Xie, J.; Zhou, Q.; Li, C.; Wang, W.; Hou, Y.; Zhang, B.; Wang, X. An Unexpected Role of the Monodentate Ligand in Photocatalytic Hydrogen Production of the Pentadentate Ligand-Based Cobalt Complexes. *Chem. Commun.* **2014**, 50 (49), 6520. https://doi.org/10.1039/c4cc01471e.
29. Varma, S.; Castillo, C. E.; Stoll, T.; Fortage, J.; Blackman, A. G.; Molton, F.; Deronzier, A.; Collomb, M.-N. Efficient Photocatalytic Hydrogen Production in Water Using a Cobalt(III) Tetraaza-Macrocyclic Catalyst: Electrochemical Generation of the Low-Valent Co(I) Species and Its Reactivity toward Proton Reduction. *Phys. Chem. Chem. Phys.* **2013**, *15* (40), 17544. https://doi.org/10.1039/c3cp52641k.
30. McCrory, C. C. L.; Uyeda, C.; Peters, J. C. Electrocatalytic Hydrogen Evolution in Acidic Water with Molecular Cobalt Tetraazamacrocycles. *J. Am. Chem. Soc.* **2012**, 134 (6), 3164–3170. https://doi.org/10.1021/ja210661k.
31. Grau, S.; Schilling, M.; Moonshiram, D.; Benet-Buchholz, J.; Luber, S.; Llobet, A.; Gimbert-Suriñach, C. Electrochemically and Photochemically Induced Hydrogen Evolution Catalysis with Cobalt Tetraazamacrocycles Occurs Through Different Pathways. *ChemSusChem* **2020**, 13 (10), 2745–2752. https://doi.org/10.1002/cssc.202000283.
32. Singh, W. M.; Baine, T.; Kudo, S.; Tian, S.; Ma, X. A. N.; Zhou, H.; Deyonker, N. J.; Pham, T. C.; Bollinger, J. C.; Baker, D. L.; et al. Electrocatalytic and Photocatalytic Hydrogen Production in Aqueous Solution by a Molecular Cobalt Complex. *Angew. Chem. Int. Ed.* **2012**, 51 (24), 5941–5944. https://doi.org/10.1002/anie.201200082.
33. Wang, P.; Liang, G.; Reddy, M. R.; Long, M.; Driskill, K.; Lyons, C.; Donnadieu, B.; Bollinger, J. C.; Webster, C. E.; Zhao, X. Electronic and Steric Tuning of Catalytic H_2 Evolution by Cobalt Complexes with Pentadentate Polypyridyl-Amine Ligands. *J. Am. Chem. Soc.* **2018**, 140 (29), 9219–9229. https://doi.org/10.1021/jacs.8b05108.
34. Song, X.; Wen, H.; Ma, C.; Chen, H.; Chen, C. Hydrogen Photogeneration Catalyzed by a Cobalt Complex of a Pentadentate Aminopyridine-Based Ligand. *New J. Chem.* **2015**, 39 (3), 1734–1741. https://doi.org/10.1039/c4nj01858c.
35. Tok, G. C.; Freiberg, A. T. S.; Gasteiger, H. A.; Hess, C. R. Electrocatalytic H_2 Evolution by the Co-Mabiq Complex Requires Tempering of the Redox-Active Ligand. *ChemCatChem* **2019**, 11 (16), 3973–3981. https://doi.org/10.1002/cctc.201900953.
36. Wang, P.; Liang, G.; Smith, N.; Hill, K.; Donnadieu, B.; Webster, C. E.; Zhao, X. Enhanced Hydrogen Evolution in Neutral Water Catalyzed by a Cobalt Complex with a Softer Polypyridyl Ligand. *Angew. Chem. Int. Ed.* **2020**, 59 (31), 12694–12697. https://doi.org/10.1002/anie.202002640.
37. Basu, D.; Mazumder, S.; Shi, X.; Baydoun, H.; Niklas, J.; Poluektov, O.; Schlegel, H. B.; Verani, C. N. Ligand Transformations and Efficient Proton/Water Reduction with Cobalt Catalysts Based on Pentadentate Pyridine-Rich Environments. *Angew. Chem. Int. Ed.* **2015**, 54 (7), 2105–2110. https://doi.org/10.1002/anie.201409813.
38. Tong, L.; Kopecky, A.; Zong, R.; Gagnon, K. J.; Ahlquist, M. S. G.; Thummel, R. P. Light-Driven Proton Reduction in Aqueous Medium Catalyzed by a Family of Cobalt Complexes with Tetradentate Polypyridine-Type Ligands. *Inorg. Chem.* **2015**, 54 (16), 7873–7884. https://doi.org/10.1021/acs.inorgchem.5b00915.
39. Liu, J.; Liao, R. Z.; Heinemann, F. W.; Meyer, K.; Thummel, R. P.; Zhang, Y.; Tong, L. Electrocatalytic Hydrogen Evolution by Cobalt Complexes with a Redox Non-Innocent Polypyridine Ligand. *Inorg. Chem.* **2021**, 60 (23), 17976–17985. https://doi.org/10.1021/acs.inorgchem.1c02539.
40. Kohler, L.; Niklas, J.; Johnson, R. C.; Zeller, M.; Poluektov, O. G.; Mulfort, K. L. Molecular Cobalt Catalysts for H_2 Generation with Redox Activity and Proton Relays in the Second Coordination Sphere. *Inorg. Chem.* 2019, 58 (2), acs.inorgchem.8b03297. https://doi.org/10.1021/acs.inorgchem.8b03297.
41. Call, A.; Codolà, Z.; Acuña-Parés, F.; Lloret-Fillol, J. Photo- and Electrocatalytic H_2 Production by New First-Row Transition-Metal Complexes Based on an Aminopyridine Pentadentate Ligand. *Chem. Eur. J.* **2014**, 20 (20), 6171–6183. https://doi.org/10.1002/chem.201303317.
42. Natali, M.; Badetti, E.; Deponti, E.; Gamberoni, M.; Scaramuzzo, F. A.; Sartorel, A.; Zonta, C. Photoinduced Hydrogen Evolution with New Tetradentate Cobalt(II) Complexes Based on the TPMA Ligand. *Dalton Trans.* **2016**, 45 (37), 14764–14773. https://doi.org/10.1039/C6DT01705C.
43. Müller, P.; Probst, B.; Spingler, B.; Blacque, O.; Alberto, R. Polar Substituents Enable Efficient Catalysis for a Class of Cobalt Polypyridyl Hydrogen Evolving Catalysts. *Helv. Chim. Acta* **2022**, 105 (3). https://doi.org/10.1002/hlca.202100237.
44. Dawson, R.; Elliot, D.; Elliot, W. *Data for Biochemical Research*; Oxford Science Publ, 1986.
